# Supplementary material for: Age and Ketogenic Diet Have Dissociable Effects on Synapse-Related Gene Expression Between Hippocampal Subregions
Source: Front Aging Neurosci. 2019 Sep 13;11:239. doi: 10.3389/fnagi.2019.00239 (PMC6755342; doi:10.3389/fnagi.2019.00239)
Supplement: Supplementary file 1 [file Data_Sheet_1.docx]

**Supplementary Material for:**

**Age and ketogenic diet have dissociable effects on synapse-related gene expression between hippocampal subregions**

Abbi R. Hernandez^1^*, Caesar M. Hernandez^1^*, Leah M. Truckenbrod^1^, Keila T. Campos, Joseph A. McQuail^1^, Jennifer L. Bizon^1^, Sara N. Burke^1,2 #^

*Indicates these authors contributed equally

^1^Department of Neuroscience, University of Florida, Gainesville, FL, USA; ^2^Institute on Aging, University of Florida, Gainesville, FL, USA

^#^Indicates corresponding author:

Burkes@ufl.edu

Figure S1: Weight at time of sacrifice relative to starting weight. Weight change was significantly decreased in animals fed a ketogenic diet ( F_[1, 26]_ = 26.61; p < 0.001). Furthermore, obese aged animals that were used to overconsumption on an *ad lib* diet lost significantly more weight than young animals (F_[1, 26]_ = 11.12; p = 0.003). Boxes represent the upper and lower quartiles around the median, the whiskers are the minimum and maximum observed values and + indicates the group mean. Dotted line represents no change in weight.

| Term | Gene Count | %Genes Contributing | Genes Contributing to Enrichment | p value | FDR-adjusted p-value | Fold Enrichment |
| --- | --- | --- | --- | --- | --- | --- |
| :Glutamatergic synapse | 21 | 53.85 | GNAI1, GRIK5, GRIN2A, GRIA4, HOMER1, SHANK2, GRM1, ITPR1, SLC17A7, SLC1A2, GRM3, SLC1A3, GRM2, GNAQ, GRIA2, GRIN2B, GRIA1, GLS, DLG4, SLC38A1, PLCB1 | 6.14  E-28 | 6.63  E-25 | 37.24 |
| GABAergic synapse | 14 | 35.90 | GABRG1, GABRG2, SLC6A1, GABRA4, GNAI1, GABRA5, GABBR1, GABBR2, GPHN, GLS, ABAT, SLC38A1, GAD1, NSF | 5.01  E-17 | 5.41  E-14 | 32.81 |
| Retrograde endocannabinoid signaling | 13 | 33.33 | GABRG1, GABRG2, GABRA4, GNAI1, GABRA5, GRIA4, GRM1, ITPR1, SLC17A7, GNAQ, GRIA2, GRIA1, PLCB1 | 2.23  E-14 | 2.41  E-11 | 25.74 |
| Nicotine addiction | 10 | 25.64 | SLC17A7, GABRG1, GABRG2, GRIN2B, GRIA2, GABRA4, GRIA1, GABRA5, GRIN2A, GRIA4 | 1.11  E-13 | 1.20  E-10 | 50.98 |
| Neuroactive ligand-receptor interaction | 16 | 41.03 | GABRG1, GABRG2, GABRA4, GABRA5, GRIK5, GABBR1, GRIN2A, GABBR2, GRIA4, ADORA1, GRM1, GRM3, GRM2, GRIA2, GRIN2B, GRIA1 | 1.36  E-12 | 1.47  E-09 | 11.17 |
| Cocaine addiction | 8 | 20.51 | GRM3, CDK5R1, GRM2, GRIN2B, GRIA2, GNAI1, GRIN2A, DLG4 | 1.45  E-09 | 1.57  E-06 | 35.46 |
| Circadian entrainment | 9 | 23.08 | GNAQ, GRIN2B, GRIA2, GNAI1, GRIA1, GRIN2A, GRIA4, PLCB1, ITPR1 | 1.29  E-08 | 1.39  E-05 | 18.92 |
| Long-term potentiation | 8 | 20.51 | GNAQ, GRIN2B, GRIA2, GRIA1, GRIN2A, PLCB1, GRM1, ITPR1 | 1.77  E-08 | 1.92  E-05 | 25.10 |
| Dopaminergic synapse | 9 | 23.08 | GNAQ, GRIN2B, GRIA2, GNAI1, GRIA1, GRIN2A, GRIA4, PLCB1, ITPR1 | 1.22  E-07 | 1.32  E-04 | 14.23 |
| Morphine addiction | 8 | 20.51 | GABRG1, GABRG2, GABRA4, GNAI1, GABRA5, GABBR1, GABBR2, ADORA1 | 2.03  E-07 | 2.20  E-04 | 17.73 |
| Long-term depression | 7 | 17.95 | GNAQ, GRIA2, GNAI1, GRIA1, PLCB1, GRM1, ITPR1 | 3.93  E-07 | 4.24  E-04 | 23.02 |
| cAMP signaling pathway | 9 | 23.08 | GRIN2B, GRIA2, GNAI1, GRIA1, GABBR1, GRIN2A, GRIA4, GABBR2, ADORA1 | 2.88  E-06 | 0.00 | 9.41 |
| Estrogen signaling pathway | 7 | 17.95 | GNAQ, GNAI1, GABBR1, GABBR2, PLCB1, GRM1, ITPR1 | 5.27  E-06 | 0.01 | 14.87 |
| Amyotrophic lateral sclerosis (ALS) | 5 | 12.82 | SLC1A2, GRIN2B, GRIA2, GRIA1, GRIN2A | 1.26  E-04 | 0.14 | 18.54 |
| Alzheimer's disease | 7 | 17.95 | CDK5R1, APP, GNAQ, GRIN2B, GRIN2A, PLCB1, ITPR1 | 1.96  E-04 | 0.21 | 7.84 |
| Amphetamine addiction | 5 | 12.82 | GRIN2B, GRIA2, GRIA1, GRIN2A, GRIA4 | 2.28  E-04 | 0.25 | 15.93 |
| Renin secretion | 5 | 12.82 | GNAQ, GNAI1, PLCB1, ADORA1, ITPR1 | 2.72  E-04 | 0.29 | 15.22 |
| Alanine, aspartate and glutamate metabolism | 4 | 10.26 | ALDH5A1, GLS, ABAT, GAD1 | 5.90  E-04 | 0.64 | 23.31 |
| Gap junction | 5 | 12.82 | GNAQ, GNAI1, PLCB1, GRM1, ITPR1 | 7.70  E-04 | 0.83 | 11.59 |
| Serotonergic synapse | 5 | 12.82 | APP, GNAQ, GNAI1, PLCB1, ITPR1 | 0.00 | 2.92 | 8.22 |
| Gastric acid secretion | 4 | 10.26 | GNAQ, GNAI1, PLCB1, ITPR1 | 0.00 | 5.23 | 11.17 |
| cGMP-PKG signaling pathway | 5 | 12.82 | GNAQ, GNAI1, PLCB1, ADORA1, ITPR1 | 0.01 | 7.42 | 6.29 |
| Butanoate metabolism | 3 | 7.69 | ALDH5A1, ABAT, GAD1 | 0.01 | 8.07 | 21.85 |
| Calcium signaling pathway | 5 | 12.82 | GNAQ, GRIN2A, PLCB1, GRM1, ITPR1 | 0.01 | 11.49 | 5.51 |
| Huntington's disease | 5 | 12.82 | GNAQ, GRIN2B, DLG4, PLCB1, ITPR1 | 0.02 | 15.41 | 5.02 |
| Cholinergic synapse | 4 | 10.26 | GNAQ, GNAI1, PLCB1, ITPR1 | 0.02 | 15.98 | 7.28 |
| Inflammatory mediator regulation of TRP channels | 4 | 10.26 | GNAQ, PLA2G6, PLCB1, ITPR1 | 0.02 | 17.05 | 7.09 |
| Rap1 signaling pathway | 5 | 12.82 | GNAQ, GRIN2B, GNAI1, GRIN2A, PLCB1 | 0.02 | 18.62 | 4.72 |
| Vascular smooth muscle contraction | 4 | 10.26 | GNAQ, PLA2G6, PLCB1, ITPR1 | 0.02 | 19.66 | 6.69 |
| Sphingolipid signaling pathway | 4 | 10.26 | GNAQ, GNAI1, PLCB1, ADORA1 | 0.02 | 20.43 | 6.58 |
| Platelet activation | 4 | 10.26 | GNAQ, GNAI1, PLCB1, ITPR1 | 0.03 | 24.86 | 6.04 |
| Oxytocin signaling pathway | 4 | 10.26 | GNAQ, GNAI1, PLCB1, ITPR1 | 0.04 | 32.23 | 5.37 |
| Thyroid hormone synthesis | 3 | 7.69 | GNAQ, PLCB1, ITPR1 | 0.04 | 36.75 | 9.00 |
| Salivary secretion | 3 | 7.69 | GNAQ, PLCB1, ITPR1 | 0.05 | 43.01 | 8.05 |
| Aldosterone synthesis and secretion | 3 | 7.69 | GNAQ, PLCB1, ITPR1 | 0.06 | 49.10 | 7.28 |
| GnRH signaling pathway | 3 | 7.69 | GNAQ, PLCB1, ITPR1 | 0.07 | 54.91 | 6.65 |
| Pancreatic secretion | 3 | 7.69 | GNAQ, PLCB1, ITPR1 | 0.08 | 57.68 | 6.37 |
| Melanogenesis | 3 | 7.69 | GNAQ, GNAI1, PLCB1 | 0.08 | 59.70 | 6.18 |
| Glucagon signaling pathway | 3 | 7.69 | GNAQ, PLCB1, ITPR1 | 0.08 | 60.36 | 6.12 |
| Chagas disease (American trypanosomiasis) | 3 | 7.69 | GNAQ, GNAI1, PLCB1 | 0.09 | 64.81 | 5.72 |

Table S1: Pathways significantly associated with the gene expression changes observed within the dentate gyrus of rats on a ketogenic diet as identified by DAVID.

| Term | Gene Count | %Genes Contributing | Genes Contributing to Enrichment | p value | FDR-adjusted p-value | Fold Enrichment |
| --- | --- | --- | --- | --- | --- | --- |
| Glutamatergic synapse | 12 | 0.31 | GNAQ, GRIN2B, GNAI1, GRIK2, GRM7, GLS, GRIK4, GRIN1, GRIN2A, HOMER1, SHANK2, ITPR1 | 2.87  E-15 | 2.98  E-12 | 35.16 |
| GABAergic synapse | 9 | 0.23 | GPHN, GABRG3, GABRB3, SLC6A1, GNAI1, GLS, GABRA5, GABBR2, NSF | 5.12  E-11 | 5.28  E-08 | 34.85 |
| Neuroactive ligand-receptor interaction | 10 | 0.26 | GABRG3, GABRB3, GRIN2B, GRIK2, GRM7, GRIK4, GRIN1, GABRA5, GRIN2A, GABBR2 | 4.38  E-08 | 4.52  E-05 | 11.54 |
| Nicotine addiction | 6 | 0.15 | GABRG3, GABRB3, GRIN2B, GRIN1, GABRA5, GRIN2A | 6.99  E-08 | 7.21  E-05 | 50.54 |
| :Alzheimer's disease | 8 | 0.21 | CDK5R1, APP, GNAQ, GRIN2B, GRIN1, SNCA, GRIN2A, ITPR1 | 4.45  E-07 | 4.59  E-04 | 14.81 |
| Circadian entrainment | 6 | 0.15 | GNAQ, GRIN2B, GNAI1, GRIN1, GRIN2A, ITPR1 | 6.16  E-06 | 0.01 | 20.84 |
| Cocaine addiction | 5 | 0.13 | CDK5R1, GRIN2B, GNAI1, GRIN1, GRIN2A | 7.35  E-06 | 0.01 | 36.62 |
| Retrograde endocannabinoid signaling | 6 | 0.15 | GABRG3, GNAQ, GABRB3, GNAI1, GABRA5, ITPR1 | 8.28  E-06 | 0.01 | 19.63 |
| Long-term potentiation | 5 | 0.13 | GNAQ, GRIN2B, GRIN1, GRIN2A, ITPR1 | 2.94  E-05 | 0.03 | 25.92 |
| Morphine addiction | 5 | 0.13 | GABRG3, GABRB3, GNAI1, GABRA5, GABBR2 | 1.16  E-04 | 0.12 | 18.31 |
| Serotonergic synapse | 5 | 0.13 | APP, GNAQ, GABRB3, GNAI1, ITPR1 | 3.66  E-04 | 0.38 | 13.59 |
| Dopaminergic synapse | 5 | 0.13 | GNAQ, GRIN2B, GNAI1, GRIN2A, ITPR1 | 4.25  E-04 | 0.44 | 13.06 |
| cAMP signaling pathway | 5 | 0.13 | GRIN2B, GNAI1, GRIN1, GRIN2A, GABBR2 | 0.00 | 2.04 | 8.64 |
| Estrogen signaling pathway | 4 | 0.10 | GNAQ, GNAI1, GABBR2, ITPR1 | 0.00 | 2.44 | 14.04 |
| Rap1 signaling pathway | 5 | 0.13 | GNAQ, GRIN2B, GNAI1, GRIN1, GRIN2A | 0.00 | 2.95 | 7.80 |
| Amyotrophic lateral sclerosis (ALS) | 3 | 0.08 | GRIN2B, GRIN1, GRIN2A | 0.01 | 10.26 | 18.38 |
| Alcoholism | 4 | 0.10 | GRIN2B, GNAI1, GRIN1, GRIN2A | 0.01 | 12.55 | 7.66 |
| Long-term depression | 3 | 0.08 | GNAQ, GNAI1, ITPR1 | 0.01 | 12.75 | 16.30 |
| Amphetamine addiction | 3 | 0.08 | GRIN2B, GRIN1, GRIN2A | 0.01 | 13.49 | 15.79 |
| Calcium signaling pathway | 4 | 0.10 | GNAQ, GRIN1, GRIN2A, ITPR1 | 0.01 | 14.23 | 7.28 |
| Renin secretion | 3 | 0.08 | GNAQ, GNAI1, ITPR1 | 0.02 | 14.64 | 15.09 |
| Gastric acid secretion | 3 | 0.08 | GNAQ, GNAI1, ITPR1 | 0.02 | 17.01 | 13.85 |
| Huntington's disease | 4 | 0.10 | GNAQ, GRIN2B, GRIN1, ITPR1 | 0.02 | 17.87 | 6.64 |
| Gap junction | 3 | 0.08 | GNAQ, GNAI1, ITPR1 | 0.03 | 23.33 | 11.49 |
| Cholinergic synapse | 3 | 0.08 | GNAQ, GNAI1, ITPR1 | 0.04 | 34.11 | 9.02 |
| Platelet activation | 3 | 0.08 | GNAQ, GNAI1, ITPR1 | 0.06 | 44.50 | 7.49 |
| Oxytocin signaling pathway | 3 | 0.08 | GNAQ, GNAI1, ITPR1 | 0.07 | 51.84 | 6.65 |
| cGMP-PKG signaling pathway | 3 | 0.08 | GNAQ, GNAI1, ITPR1 | 0.08 | 55.96 | 6.24 |

Table S2: Pathways significantly associated with the gene expression changes observed within CA3 of aged rats as identified by DAVID.

| Gene | Age | Diet | Mean (Z-Scores) | Std. Deviation | N |
| --- | --- | --- | --- | --- | --- |
| Abat | Young | Standard | 0.966 | 0.823 | 7 |
|  |  | Ketogenic | -0.632 | 0.834 | 7 |
|  |  | Total | 0.167 | 1.150 | 14 |
|  | Aged | Standard | 0.179 | 0.596 | 8 |
|  |  | Ketogenic | -0.942 | 0.618 | 4 |
|  |  | Total | -0.195 | 0.796 | 12 |
|  | Total | Standard | 0.546 | 0.795 | 15 |
|  |  | Ketogenic | -0.745 | 0.746 | 11 |
|  |  | Total | 0.000 | 1.000 | 26 |
| Adora1 | Young | Standard | 0.927 | 0.654 | 7 |
|  |  | Ketogenic | -0.602 | 0.895 | 7 |
|  |  | Total | 0.162 | 1.094 | 14 |
|  | Aged | Standard | 0.163 | 0.753 | 8 |
|  |  | Ketogenic | -0.895 | 0.750 | 4 |
|  |  | Total | -0.190 | 0.886 | 12 |
|  | Total | Standard | 0.520 | 0.789 | 15 |
|  |  | Ketogenic | -0.709 | 0.819 | 11 |
|  |  | Total | 0.000 | 1.000 | 26 |
| Aldh5a1 | Young | Standard | 0.813 | 0.670 | 7 |
|  |  | Ketogenic | -0.627 | 0.983 | 7 |
|  |  | Total | 0.093 | 1.101 | 14 |
|  | Aged | Standard | 0.208 | 0.723 | 8 |
|  |  | Ketogenic | -0.741 | 0.985 | 4 |
|  |  | Total | -0.109 | 0.903 | 12 |
|  | Total | Standard | 0.490 | 0.743 | 15 |
|  |  | Ketogenic | -0.669 | 0.935 | 11 |
|  |  | Total | 0.000 | 1.000 | 26 |
| App | Young | Standard | 0.756 | 0.537 | 7 |
|  |  | Ketogenic | -0.773 | 0.962 | 7 |
|  |  | Total | -0.008 | 1.091 | 14 |
|  | Aged | Standard | 0.299 | 0.774 | 8 |
|  |  | Ketogenic | -0.569 | 1.052 | 4 |
|  |  | Total | 0.010 | 0.930 | 12 |
|  | Total | Standard | 0.512 | 0.692 | 15 |
|  |  | Ketogenic | -0.699 | 0.948 | 11 |
|  |  | Total | 0.000 | 1.000 | 26 |
| Bdnf | Young | Standard | 0.020 | 0.648 | 7 |
|  |  | Ketogenic | -0.674 | 0.691 | 7 |
|  |  | Total | -0.327 | 0.737 | 14 |
|  | Aged | Standard | 0.563 | 0.861 | 8 |
|  |  | Ketogenic | 0.020 | 1.703 | 4 |
|  |  | Total | 0.382 | 1.155 | 12 |
|  | Total | Standard | 0.309 | 0.793 | 15 |
|  |  | Ketogenic | -0.422 | 1.131 | 11 |
|  |  | Total | 0.000 | 1.000 | 26 |
| Cacna1a | Young | Standard | 0.768 | 1.211 | 7 |
|  |  | Ketogenic | -0.610 | 0.856 | 7 |
|  |  | Total | 0.079 | 1.235 | 14 |
|  | Aged | Standard | 0.103 | 0.632 | 8 |
|  |  | Ketogenic | -0.482 | 0.650 | 4 |
|  |  | Total | -0.092 | 0.672 | 12 |
|  | Total | Standard | 0.413 | 0.973 | 15 |
|  |  | Ketogenic | -0.563 | 0.755 | 11 |
|  |  | Total | 0.000 | 1.000 | 26 |
| Cdk5r1 | Young | Standard | 0.836 | 1.191 | 7 |
|  |  | Ketogenic | -0.668 | 0.628 | 7 |
|  |  | Total | 0.084 | 1.202 | 14 |
|  | Aged | Standard | 0.254 | 0.531 | 8 |
|  |  | Ketogenic | -0.802 | 0.590 | 4 |
|  |  | Total | -0.098 | 0.738 | 12 |
|  | Total | Standard | 0.526 | 0.916 | 15 |
|  |  | Ketogenic | -0.717 | 0.588 | 11 |
|  |  | Total | 0.000 | 1.000 | 26 |
| Dlg4 | Young | Standard | 0.877 | 0.662 | 7 |
|  |  | Ketogenic | -0.527 | 1.005 | 7 |
|  |  | Total | 0.175 | 1.095 | 14 |
|  | Aged | Standard | 0.210 | 0.602 | 8 |
|  |  | Ketogenic | -1.033 | 0.780 | 4 |
|  |  | Total | -0.204 | 0.878 | 12 |
|  | Total | Standard | 0.521 | 0.699 | 15 |
|  |  | Ketogenic | -0.711 | 0.924 | 11 |
|  |  | Total | 0.000 | 1.000 | 26 |
| Gabbr1 | Young | Standard | 0.843 | 0.873 | 7 |
|  |  | Ketogenic | -0.549 | 0.993 | 7 |
|  |  | Total | 0.147 | 1.153 | 14 |
|  | Aged | Standard | 0.202 | 0.622 | 8 |
|  |  | Ketogenic | -0.920 | 0.578 | 4 |
|  |  | Total | -0.172 | 0.802 | 12 |
|  | Total | Standard | 0.501 | 0.794 | 15 |
|  |  | Ketogenic | -0.684 | 0.853 | 11 |
|  |  | Total | 0.000 | 1.000 | 26 |
| Gabbr2 | Young | Standard | 0.757 | 0.653 | 7 |
|  |  | Ketogenic | -0.620 | 1.081 | 7 |
|  |  | Total | 0.068 | 1.117 | 14 |
|  | Aged | Standard | 0.313 | 0.627 | 8 |
|  |  | Ketogenic | -0.865 | 0.854 | 4 |
|  |  | Total | -0.079 | 0.887 | 12 |
|  | Total | Standard | 0.520 | 0.657 | 15 |
|  |  | Ketogenic | -0.709 | 0.967 | 11 |
|  |  | Total | 0.000 | 1.000 | 26 |
| Gabra1 | Young | Standard | -0.141 | 0.396 | 7 |
|  |  | Ketogenic | -0.336 | 1.182 | 7 |
|  |  | Total | -0.239 | 0.853 | 14 |
|  | Aged | Standard | 0.444 | 0.821 | 8 |
|  |  | Ketogenic | -0.053 | 1.678 | 4 |
|  |  | Total | 0.278 | 1.121 | 12 |
|  | Total | Standard | 0.171 | 0.704 | 15 |
|  |  | Ketogenic | -0.233 | 1.305 | 11 |
|  |  | Total | 0.000 | 1.000 | 26 |
| Gabra2 | Young | Standard | 0.675 | 0.482 | 7 |
|  |  | Ketogenic | -0.577 | 1.051 | 7 |
|  |  | Total | 0.049 | 1.019 | 14 |
|  | Aged | Standard | 0.195 | 0.812 | 8 |
|  |  | Ketogenic | -0.562 | 1.327 | 4 |
|  |  | Total | -0.057 | 1.019 | 12 |
|  | Total | Standard | 0.419 | 0.701 | 15 |
|  |  | Ketogenic | -0.571 | 1.091 | 11 |
|  |  | Total | 0.000 | 1.000 | 26 |
| Gabra4 | Young | Standard | 0.808 | 0.744 | 7 |
|  |  | Ketogenic | -0.645 | 0.990 | 7 |
|  |  | Total | 0.082 | 1.130 | 14 |
|  | Aged | Standard | 0.224 | 0.617 | 8 |
|  |  | Ketogenic | -0.734 | 1.016 | 4 |
|  |  | Total | -0.095 | 0.864 | 12 |
|  | Total | Standard | 0.497 | 0.720 | 15 |
|  |  | Ketogenic | -0.677 | 0.949 | 11 |
|  |  | Total | 0.000 | 1.000 | 26 |
| Gabra5 | Young | Standard | 0.816 | 0.755 | 7 |
|  |  | Ketogenic | -0.572 | 0.832 | 7 |
|  |  | Total | 0.122 | 1.049 | 14 |
|  | Aged | Standard | 0.218 | 0.750 | 8 |
|  |  | Ketogenic | -0.863 | 1.030 | 4 |
|  |  | Total | -0.142 | 0.965 | 12 |
|  | Total | Standard | 0.497 | 0.788 | 15 |
|  |  | Ketogenic | -0.678 | 0.869 | 11 |
|  |  | Total | 0.000 | 1.000 | 26 |
| Gabrb1 | Young | Standard | 0.137 | 0.666 | 7 |
|  |  | Ketogenic | -0.249 | 1.231 | 7 |
|  |  | Total | -0.056 | 0.972 | 14 |
|  | Aged | Standard | 0.347 | 0.915 | 8 |
|  |  | Ketogenic | -0.498 | 1.273 | 4 |
|  |  | Total | 0.065 | 1.072 | 12 |
|  | Total | Standard | 0.249 | 0.788 | 15 |
|  |  | Ketogenic | -0.339 | 1.188 | 11 |
|  |  | Total | 0.000 | 1.000 | 26 |
| Gabrb3 | Young | Standard | 0.515 | 1.006 | 7 |
|  |  | Ketogenic | -0.628 | 0.817 | 7 |
|  |  | Total | -0.056 | 1.062 | 14 |
|  | Aged | Standard | 0.444 | 0.681 | 8 |
|  |  | Ketogenic | -0.691 | 1.091 | 4 |
|  |  | Total | 0.066 | 0.965 | 12 |
|  | Total | Standard | 0.477 | 0.816 | 15 |
|  |  | Ketogenic | -0.651 | 0.871 | 11 |
|  |  | Total | 0.000 | 1.000 | 26 |
| Gabrd | Young | Standard | 0.849 | 1.306 | 7 |
|  |  | Ketogenic | -0.632 | 0.755 | 7 |
|  |  | Total | 0.109 | 1.281 | 14 |
|  | Aged | Standard | 0.092 | 0.386 | 8 |
|  |  | Ketogenic | -0.564 | 0.608 | 4 |
|  |  | Total | -0.127 | 0.548 | 12 |
|  | Total | Standard | 0.445 | 0.979 | 15 |
|  |  | Ketogenic | -0.607 | 0.674 | 11 |
|  |  | Total | 0.000 | 1.000 | 26 |
| Gabrg1 | Young | Standard | 0.953 | 0.678 | 7 |
|  |  | Ketogenic | -0.671 | 0.731 | 7 |
|  |  | Total | 0.141 | 1.081 | 14 |
|  | Aged | Standard | 0.229 | 0.618 | 8 |
|  |  | Ketogenic | -0.953 | 0.966 | 4 |
|  |  | Total | -0.165 | 0.914 | 12 |
|  | Total | Standard | 0.567 | 0.727 | 15 |
|  |  | Ketogenic | -0.773 | 0.788 | 11 |
|  |  | Total | 0.000 | 1.000 | 26 |
| Gabrg2 | Young | Standard | 0.789 | 0.486 | 7 |
|  |  | Ketogenic | -0.639 | 0.902 | 7 |
|  |  | Total | 0.075 | 1.017 | 14 |
|  | Aged | Standard | 0.213 | 0.841 | 8 |
|  |  | Ketogenic | -0.689 | 1.194 | 4 |
|  |  | Total | -0.088 | 1.018 | 12 |
|  | Total | Standard | 0.482 | 0.737 | 15 |
|  |  | Ketogenic | -0.657 | 0.957 | 11 |
|  |  | Total | 0.000 | 1.000 | 26 |
| Gad1 | Young | Standard | 0.872 | 0.627 | 7 |
|  |  | Ketogenic | -0.636 | 1.051 | 7 |
|  |  | Total | 0.118 | 1.142 | 14 |
|  | Aged | Standard | 0.133 | 0.686 | 8 |
|  |  | Ketogenic | -0.678 | 0.928 | 4 |
|  |  | Total | -0.137 | 0.833 | 12 |
|  | Total | Standard | 0.478 | 0.741 | 15 |
|  |  | Ketogenic | -0.651 | 0.960 | 11 |
|  |  | Total | 0.000 | 1.000 | 26 |
| Gls | Young | Standard | 0.948 | 0.865 | 7 |
|  |  | Ketogenic | -0.741 | 0.793 | 7 |
|  |  | Total | 0.103 | 1.184 | 14 |
|  | Aged | Standard | 0.102 | 0.696 | 8 |
|  |  | Ketogenic | -0.566 | 0.787 | 4 |
|  |  | Total | -0.121 | 0.765 | 12 |
|  | Total | Standard | 0.497 | 0.868 | 15 |
|  |  | Ketogenic | -0.677 | 0.756 | 11 |
|  |  | Total | 0.000 | 1.000 | 26 |
| Glul | Young | Standard | -0.180 | 0.909 | 7 |
|  |  | Ketogenic | -0.214 | 0.839 | 7 |
|  |  | Total | -0.197 | 0.840 | 14 |
|  | Aged | Standard | 0.651 | 0.871 | 8 |
|  |  | Ketogenic | -0.612 | 1.300 | 4 |
|  |  | Total | 0.230 | 1.154 | 12 |
|  | Total | Standard | 0.263 | 0.958 | 15 |
|  |  | Ketogenic | -0.359 | 0.985 | 11 |
|  |  | Total | 0.000 | 1.000 | 26 |
| Gnai1 | Young | Standard | 0.555 | 0.840 | 7 |
|  |  | Ketogenic | -0.720 | 0.769 | 7 |
|  |  | Total | -0.083 | 1.018 | 14 |
|  | Aged | Standard | 0.470 | 0.907 | 8 |
|  |  | Ketogenic | -0.651 | 0.859 | 4 |
|  |  | Total | 0.096 | 1.014 | 12 |
|  | Total | Standard | 0.510 | 0.846 | 15 |
|  |  | Ketogenic | -0.695 | 0.760 | 11 |
|  |  | Total | 0.000 | 1.000 | 26 |
| Gnaq | Young | Standard | 0.645 | 1.058 | 7 |
|  |  | Ketogenic | -0.694 | 0.774 | 7 |
|  |  | Total | -0.024 | 1.130 | 14 |
|  | Aged | Standard | 0.418 | 0.506 | 8 |
|  |  | Ketogenic | -1.001 | 0.778 | 3 |
|  |  | Total | 0.031 | 0.860 | 11 |
|  | Total | Standard | 0.524 | 0.788 | 15 |
|  |  | Ketogenic | -0.786 | 0.746 | 10 |
|  |  | Total | 0.000 | 1.000 | 25 |
| Gphn | Young | Standard | 0.686 | 1.122 | 7 |
|  |  | Ketogenic | -0.710 | 0.711 | 7 |
|  |  | Total | -0.012 | 1.158 | 14 |
|  | Aged | Standard | 0.349 | 0.656 | 8 |
|  |  | Ketogenic | -0.657 | 0.789 | 4 |
|  |  | Total | 0.014 | 0.830 | 12 |
|  | Total | Standard | 0.507 | 0.886 | 15 |
|  |  | Ketogenic | -0.691 | 0.701 | 11 |
|  |  | Total | 0.000 | 1.000 | 26 |
| Gria1 | Young | Standard | 1.005 | 0.593 | 7 |
|  |  | Ketogenic | -0.553 | 1.001 | 7 |
|  |  | Total | 0.226 | 1.130 | 14 |
|  | Aged | Standard | 0.010 | 0.592 | 8 |
|  |  | Ketogenic | -0.811 | 0.930 | 4 |
|  |  | Total | -0.264 | 0.789 | 12 |
|  | Total | Standard | 0.474 | 0.768 | 15 |
|  |  | Ketogenic | -0.647 | 0.937 | 11 |
|  |  | Total | 0.000 | 1.000 | 26 |
| Gria2 | Young | Standard | 0.942 | 0.525 | 7 |
|  |  | Ketogenic | -0.739 | 0.938 | 7 |
|  |  | Total | 0.101 | 1.138 | 14 |
|  | Aged | Standard | 0.084 | 0.764 | 8 |
|  |  | Ketogenic | -0.523 | 0.964 | 4 |
|  |  | Total | -0.118 | 0.845 | 12 |
|  | Total | Standard | 0.485 | 0.779 | 15 |
|  |  | Ketogenic | -0.661 | 0.905 | 11 |
|  |  | Total | 0.000 | 1.000 | 26 |
| Gria3 | Young | Standard | 0.853 | 0.535 | 7 |
|  |  | Ketogenic | -0.576 | 0.984 | 7 |
|  |  | Total | 0.138 | 1.062 | 14 |
|  | Aged | Standard | 0.105 | 0.831 | 8 |
|  |  | Ketogenic | -0.694 | 1.035 | 4 |
|  |  | Total | -0.161 | 0.941 | 12 |
|  | Total | Standard | 0.454 | 0.785 | 15 |
|  |  | Ketogenic | -0.619 | 0.952 | 11 |
|  |  | Total | 0.000 | 1.000 | 26 |
| Gria4 | Young | Standard | 0.823 | 0.667 | 7 |
|  |  | Ketogenic | -0.781 | 0.909 | 7 |
|  |  | Total | 0.021 | 1.131 | 14 |
|  | Aged | Standard | 0.222 | 0.653 | 8 |
|  |  | Ketogenic | -0.518 | 1.142 | 4 |
|  |  | Total | -0.024 | 0.872 | 12 |
|  | Total | Standard | 0.503 | 0.707 | 15 |
|  |  | Ketogenic | -0.685 | 0.951 | 11 |
|  |  | Total | 0.000 | 1.000 | 26 |
| Grik1 | Young | Standard | 0.608 | 0.855 | 7 |
|  |  | Ketogenic | -0.708 | 1.001 | 7 |
|  |  | Total | -0.050 | 1.126 | 14 |
|  | Aged | Standard | 0.294 | 0.753 | 8 |
|  |  | Ketogenic | -0.415 | 1.023 | 4 |
|  |  | Total | 0.058 | 0.877 | 12 |
|  | Total | Standard | 0.441 | 0.790 | 15 |
|  |  | Ketogenic | -0.601 | 0.968 | 11 |
|  |  | Total | 0.000 | 1.000 | 26 |
| Grik2 | Young | Standard | 0.602 | 0.606 | 7 |
|  |  | Ketogenic | -0.596 | 1.056 | 7 |
|  |  | Total | 0.003 | 1.034 | 14 |
|  | Aged | Standard | 0.309 | 0.771 | 8 |
|  |  | Ketogenic | -0.627 | 1.237 | 4 |
|  |  | Total | -0.003 | 1.004 | 12 |
|  | Total | Standard | 0.445 | 0.691 | 15 |
|  |  | Ketogenic | -0.607 | 1.062 | 11 |
|  |  | Total | 0.000 | 1.000 | 26 |
| Grik4 | Young | Standard | 0.786 | 0.824 | 7 |
|  |  | Ketogenic | -0.623 | 0.891 | 7 |
|  |  | Total | 0.082 | 1.102 | 14 |
|  | Aged | Standard | 0.168 | 0.883 | 8 |
|  |  | Ketogenic | -0.621 | 0.796 | 4 |
|  |  | Total | -0.095 | 0.905 | 12 |
|  | Total | Standard | 0.456 | 0.885 | 15 |
|  |  | Ketogenic | -0.622 | 0.816 | 11 |
|  |  | Total | 0.000 | 1.000 | 26 |
| Grik5 | Young | Standard | 0.871 | 0.939 | 7 |
|  |  | Ketogenic | -0.620 | 0.962 | 7 |
|  |  | Total | 0.125 | 1.197 | 14 |
|  | Aged | Standard | 0.115 | 0.707 | 8 |
|  |  | Ketogenic | -0.669 | 0.501 | 4 |
|  |  | Total | -0.146 | 0.732 | 12 |
|  | Total | Standard | 0.468 | 0.883 | 15 |
|  |  | Ketogenic | -0.638 | 0.794 | 11 |
|  |  | Total | 0.000 | 1.000 | 26 |
| Grin1 | Young | Standard | 0.547 | 0.747 | 7 |
|  |  | Ketogenic | -0.481 | 1.174 | 7 |
|  |  | Total | 0.033 | 1.085 | 14 |
|  | Aged | Standard | 0.033 | 0.987 | 8 |
|  |  | Ketogenic | -0.181 | 0.950 | 4 |
|  |  | Total | -0.038 | 0.937 | 12 |
|  | Total | Standard | 0.273 | 0.893 | 15 |
|  |  | Ketogenic | -0.372 | 1.059 | 11 |
|  |  | Total | 0.000 | 1.000 | 26 |
| Grin2a | Young | Standard | 0.789 | 0.843 | 7 |
|  |  | Ketogenic | -0.577 | 0.952 | 7 |
|  |  | Total | 0.106 | 1.117 | 14 |
|  | Aged | Standard | 0.222 | 0.597 | 8 |
|  |  | Ketogenic | -0.814 | 1.012 | 4 |
|  |  | Total | -0.123 | 0.875 | 12 |
|  | Total | Standard | 0.486 | 0.754 | 15 |
|  |  | Ketogenic | -0.663 | 0.930 | 11 |
|  |  | Total | 0.000 | 1.000 | 26 |
| Grin2b | Young | Standard | 0.768 | 1.039 | 7 |
|  |  | Ketogenic | -0.676 | 0.814 | 7 |
|  |  | Total | 0.046 | 1.169 | 14 |
|  | Aged | Standard | 0.291 | 0.693 | 8 |
|  |  | Ketogenic | -0.746 | 0.563 | 4 |
|  |  | Total | -0.054 | 0.808 | 12 |
|  | Total | Standard | 0.514 | 0.874 | 15 |
|  |  | Ketogenic | -0.701 | 0.703 | 11 |
|  |  | Total | 0.000 | 1.000 | 26 |
| Grm1 | Young | Standard | 0.980 | 0.660 | 7 |
|  |  | Ketogenic | -0.541 | 0.958 | 7 |
|  |  | Total | 0.220 | 1.117 | 14 |
|  | Aged | Standard | 0.037 | 0.711 | 8 |
|  |  | Ketogenic | -0.844 | 0.755 | 4 |
|  |  | Total | -0.256 | 0.816 | 12 |
|  | Total | Standard | 0.477 | 0.823 | 15 |
|  |  | Ketogenic | -0.651 | 0.863 | 11 |
|  |  | Total | 0.000 | 1.000 | 26 |
| Grm2 | Young | Standard | 0.980 | 0.519 | 7 |
|  |  | Ketogenic | -0.635 | 1.114 | 7 |
|  |  | Total | 0.173 | 1.183 | 14 |
|  | Aged | Standard | 0.059 | 0.654 | 8 |
|  |  | Ketogenic | -0.723 | 0.652 | 4 |
|  |  | Total | -0.201 | 0.733 | 12 |
|  | Total | Standard | 0.489 | 0.745 | 15 |
|  |  | Ketogenic | -0.667 | 0.935 | 11 |
|  |  | Total | 0.000 | 1.000 | 26 |
| Grm3 | Young | Standard | 0.653 | 0.567 | 7 |
|  |  | Ketogenic | -0.708 | 0.875 | 7 |
|  |  | Total | -0.028 | 1.001 | 14 |
|  | Aged | Standard | 0.420 | 0.824 | 8 |
|  |  | Ketogenic | -0.743 | 1.095 | 4 |
|  |  | Total | 0.032 | 1.043 | 12 |
|  | Total | Standard | 0.529 | 0.702 | 15 |
|  |  | Ketogenic | -0.721 | 0.905 | 11 |
|  |  | Total | 0.000 | 1.000 | 26 |
| Grm5 | Young | Standard | 0.880 | 0.866 | 7 |
|  |  | Ketogenic | -0.574 | 1.060 | 7 |
|  |  | Total | 0.153 | 1.197 | 14 |
|  | Aged | Standard | 0.059 | 0.668 | 8 |
|  |  | Ketogenic | -0.654 | 0.624 | 4 |
|  |  | Total | -0.178 | 0.717 | 12 |
|  | Total | Standard | 0.442 | 0.851 | 15 |
|  |  | Ketogenic | -0.603 | 0.891 | 11 |
|  |  | Total | 0.000 | 1.000 | 26 |
| Grm7 | Young | Standard | 0.590 | 0.556 | 7 |
|  |  | Ketogenic | -0.565 | 1.117 | 7 |
|  |  | Total | 0.012 | 1.038 | 14 |
|  | Aged | Standard | 0.315 | 0.812 | 8 |
|  |  | Ketogenic | -0.673 | 1.121 | 4 |
|  |  | Total | -0.014 | 0.999 | 12 |
|  | Total | Standard | 0.443 | 0.694 | 15 |
|  |  | Ketogenic | -0.604 | 1.062 | 11 |
|  |  | Total | 0.000 | 1.000 | 26 |
| Homer1 | Young | Standard | 0.902 | 0.772 | 7 |
|  |  | Ketogenic | -0.756 | 0.979 | 7 |
|  |  | Total | 0.073 | 1.207 | 14 |
|  | Aged | Standard | 0.224 | 0.530 | 8 |
|  |  | Ketogenic | -0.702 | 0.739 | 4 |
|  |  | Total | -0.085 | 0.732 | 12 |
|  | Total | Standard | 0.540 | 0.720 | 15 |
|  |  | Ketogenic | -0.736 | 0.860 | 11 |
|  |  | Total | 0.000 | 1.000 | 26 |
| Itpr1 | Young | Standard | 0.655 | 1.216 | 7 |
|  |  | Ketogenic | -0.743 | 0.598 | 7 |
|  |  | Total | -0.044 | 1.172 | 14 |
|  | Aged | Standard | 0.420 | 0.621 | 8 |
|  |  | Ketogenic | -0.688 | 0.611 | 4 |
|  |  | Total | 0.051 | 0.803 | 12 |
|  | Total | Standard | 0.530 | 0.917 | 15 |
|  |  | Ketogenic | -0.723 | 0.572 | 11 |
|  |  | Total | 0.000 | 1.000 | 26 |
| Mapk1 | Young | Standard | 0.774 | 0.451 | 7 |
|  |  | Ketogenic | -0.697 | 1.141 | 7 |
|  |  | Total | 0.038 | 1.130 | 14 |
|  | Aged | Standard | 0.111 | 0.811 | 8 |
|  |  | Ketogenic | -0.355 | 1.028 | 4 |
|  |  | Total | -0.044 | 0.872 | 12 |
|  | Total | Standard | 0.420 | 0.730 | 15 |
|  |  | Ketogenic | -0.573 | 1.062 | 11 |
|  |  | Total | 0.000 | 1.000 | 26 |
| Nsf | Young | Standard | 1.022 | 0.642 | 7 |
|  |  | Ketogenic | -0.610 | 0.978 | 7 |
|  |  | Total | 0.206 | 1.162 | 14 |
|  | Aged | Standard | 0.035 | 0.650 | 8 |
|  |  | Ketogenic | -0.791 | 0.682 | 4 |
|  |  | Total | -0.240 | 0.749 | 12 |
|  | Total | Standard | 0.496 | 0.805 | 15 |
|  |  | Ketogenic | -0.676 | 0.850 | 11 |
|  |  | Total | 0.000 | 1.000 | 26 |
| P2rx7 | Young | Standard | 0.476 | 0.727 | 7 |
|  |  | Ketogenic | -0.738 | 0.862 | 7 |
|  |  | Total | -0.131 | 0.992 | 14 |
|  | Aged | Standard | 0.387 | 0.903 | 8 |
|  |  | Ketogenic | -0.316 | 1.248 | 4 |
|  |  | Total | 0.153 | 1.031 | 12 |
|  | Total | Standard | 0.429 | 0.798 | 15 |
|  |  | Ketogenic | -0.584 | 0.979 | 11 |
|  |  | Total | 0.000 | 1.000 | 26 |
| Phgdh | Young | Standard | 0.980 | 0.917 | 7 |
|  |  | Ketogenic | -0.628 | 0.948 | 7 |
|  |  | Total | 0.176 | 1.225 | 14 |
|  | Aged | Standard | 0.040 | 0.525 | 8 |
|  |  | Ketogenic | -0.697 | 0.628 | 4 |
|  |  | Total | -0.206 | 0.644 | 12 |
|  | Total | Standard | 0.479 | 0.857 | 15 |
|  |  | Ketogenic | -0.653 | 0.812 | 11 |
|  |  | Total | 0.000 | 1.000 | 26 |
| Pla2g6 | Young | Standard | 0.909 | 0.783 | 7 |
|  |  | Ketogenic | -0.527 | 0.958 | 7 |
|  |  | Total | 0.191 | 1.123 | 14 |
|  | Aged | Standard | 0.099 | 0.690 | 8 |
|  |  | Ketogenic | -0.866 | 0.746 | 4 |
|  |  | Total | -0.223 | 0.825 | 12 |
|  | Total | Standard | 0.477 | 0.822 | 15 |
|  |  | Ketogenic | -0.650 | 0.865 | 11 |
|  |  | Total | 0.000 | 1.000 | 26 |
| Plcb1 | Young | Standard | 0.886 | 0.589 | 7 |
|  |  | Ketogenic | -0.719 | 1.056 | 7 |
|  |  | Total | 0.083 | 1.170 | 14 |
|  | Aged | Standard | 0.153 | 0.642 | 8 |
|  |  | Ketogenic | -0.598 | 0.933 | 4 |
|  |  | Total | -0.097 | 0.798 | 12 |
|  | Total | Standard | 0.495 | 0.706 | 15 |
|  |  | Ketogenic | -0.675 | 0.966 | 11 |
|  |  | Total | 0.000 | 1.000 | 26 |
| Prodh | Young | Standard | 0.517 | 0.640 | 7 |
|  |  | Ketogenic | -0.551 | 1.255 | 7 |
|  |  | Total | -0.017 | 1.106 | 14 |
|  | Aged | Standard | 0.460 | 0.416 | 8 |
|  |  | Ketogenic | -0.859 | 1.038 | 4 |
|  |  | Total | 0.020 | 0.909 | 12 |
|  | Total | Standard | 0.486 | 0.513 | 15 |
|  |  | Ketogenic | -0.663 | 1.137 | 11 |
|  |  | Total | 0.000 | 1.000 | 26 |
| Shank2 | Young | Standard | 0.798 | 0.741 | 7 |
|  |  | Ketogenic | -0.632 | 1.025 | 7 |
|  |  | Total | 0.083 | 1.136 | 14 |
|  | Aged | Standard | 0.231 | 0.705 | 8 |
|  |  | Ketogenic | -0.753 | 0.809 | 4 |
|  |  | Total | -0.097 | 0.854 | 12 |
|  | Total | Standard | 0.496 | 0.755 | 15 |
|  |  | Ketogenic | -0.676 | 0.911 | 11 |
|  |  | Total | 0.000 | 1.000 | 26 |
| Slc17a7 | Young | Standard | 0.693 | 0.998 | 7 |
|  |  | Ketogenic | -0.650 | 0.956 | 7 |
|  |  | Total | 0.021 | 1.169 | 14 |
|  | Aged | Standard | 0.326 | 0.649 | 8 |
|  |  | Ketogenic | -0.726 | 0.660 | 4 |
|  |  | Total | -0.025 | 0.810 | 12 |
|  | Total | Standard | 0.497 | 0.821 | 15 |
|  |  | Ketogenic | -0.678 | 0.825 | 11 |
|  |  | Total | 0.000 | 1.000 | 26 |
| Slc1a1 | Young | Standard | 0.318 | 0.847 | 7 |
|  |  | Ketogenic | -0.617 | 0.963 | 7 |
|  |  | Total | -0.149 | 0.997 | 14 |
|  | Aged | Standard | 0.504 | 0.856 | 8 |
|  |  | Ketogenic | -0.486 | 1.104 | 4 |
|  |  | Total | 0.174 | 1.018 | 12 |
|  | Total | Standard | 0.417 | 0.826 | 15 |
|  |  | Ketogenic | -0.569 | 0.962 | 11 |
|  |  | Total | 0.000 | 1.000 | 26 |
| Slc1a2 | Young | Standard | 0.764 | 0.473 | 7 |
|  |  | Ketogenic | -0.593 | 0.915 | 7 |
|  |  | Total | 0.085 | 0.993 | 14 |
|  | Aged | Standard | 0.331 | 0.692 | 8 |
|  |  | Ketogenic | -0.961 | 1.178 | 4 |
|  |  | Total | -0.100 | 1.043 | 12 |
|  | Total | Standard | 0.533 | 0.621 | 15 |
|  |  | Ketogenic | -0.727 | 0.976 | 11 |
|  |  | Total | 0.000 | 1.000 | 26 |
| Slc1a3 | Young | Standard | 0.979 | 0.568 | 7 |
|  |  | Ketogenic | -0.686 | 0.900 | 7 |
|  |  | Total | 0.147 | 1.127 | 14 |
|  | Aged | Standard | 0.128 | 0.696 | 8 |
|  |  | Ketogenic | -0.769 | 0.879 | 4 |
|  |  | Total | -0.171 | 0.845 | 12 |
|  | Total | Standard | 0.525 | 0.757 | 15 |
|  |  | Ketogenic | -0.716 | 0.848 | 11 |
|  |  | Total | 0.000 | 1.000 | 26 |
| Slc38a1 | Young | Standard | 0.752 | 0.628 | 7 |
|  |  | Ketogenic | -0.773 | 0.980 | 7 |
|  |  | Total | -0.010 | 1.119 | 14 |
|  | Aged | Standard | 0.348 | 0.671 | 8 |
|  |  | Ketogenic | -0.660 | 0.976 | 4 |
|  |  | Total | 0.012 | 0.890 | 12 |
|  | Total | Standard | 0.537 | 0.662 | 15 |
|  |  | Ketogenic | -0.732 | 0.930 | 11 |
|  |  | Total | 0.000 | 1.000 | 26 |
| Slc6a1 | Young | Standard | 1.179 | 0.853 | 7 |
|  |  | Ketogenic | -0.638 | 0.816 | 7 |
|  |  | Total | 0.270 | 1.238 | 14 |
|  | Aged | Standard | -0.068 | 0.291 | 8 |
|  |  | Ketogenic | -0.811 | 0.516 | 4 |
|  |  | Total | -0.315 | 0.510 | 12 |
|  | Total | Standard | 0.514 | 0.876 | 15 |
|  |  | Ketogenic | -0.701 | 0.698 | 11 |
|  |  | Total | 0.000 | 1.000 | 26 |
| Slc6a11 | Young | Standard | 0.926 | 0.822 | 7 |
|  |  | Ketogenic | -0.615 | 0.786 | 7 |
|  |  | Total | 0.155 | 1.112 | 14 |
|  | Aged | Standard | 0.208 | 0.685 | 8 |
|  |  | Ketogenic | -0.959 | 0.655 | 4 |
|  |  | Total | -0.181 | 0.864 | 12 |
|  | Total | Standard | 0.543 | 0.813 | 15 |
|  |  | Ketogenic | -0.740 | 0.728 | 11 |
|  |  | Total | 0.000 | 1.000 | 26 |
| Snca | Young | Standard | 0.422 | 0.857 | 7 |
|  |  | Ketogenic | -0.356 | 1.091 | 7 |
|  |  | Total | 0.033 | 1.025 | 14 |
|  | Aged | Standard | 0.196 | 0.837 | 8 |
|  |  | Ketogenic | -0.506 | 1.302 | 4 |
|  |  | Total | -0.038 | 1.014 | 12 |
|  | Total | Standard | 0.301 | 0.824 | 15 |
|  |  | Ketogenic | -0.411 | 1.108 | 11 |
|  |  | Total | 0.000 | 1.000 | 26 |
| Srr | Young | Standard | 0.495 | 0.963 | 7 |
|  |  | Ketogenic | -0.677 | 0.891 | 7 |
|  |  | Total | -0.091 | 1.079 | 14 |
|  | Aged | Standard | 0.429 | 0.685 | 8 |
|  |  | Ketogenic | -0.540 | 1.129 | 4 |
|  |  | Total | 0.106 | 0.935 | 12 |
|  | Total | Standard | 0.460 | 0.796 | 15 |
|  |  | Ketogenic | -0.627 | 0.930 | 11 |
|  |  | Total | 0.000 | 1.000 | 26 |

Table S3: Descriptive statistics for gene expression within the DG.

| Effect | Gene | Type III Sum of Squares | df | Mean Square | F | p-values | Partial Eta Squared | Noncent. Parameter | Observed Power^bz^ | Rank | FDR q-values 0.5% |
| --- | --- | --- | --- | --- | --- | --- | --- | --- | --- | --- | --- |
| Age | Abat | 1.821 | 1 | 1.821 | 3.376 | 0.080 | 0.133 | 3.376 | 0.420 | 6 | 0.001 |
|  | Adora1 | 1.689 | 1 | 1.689 | 2.853 | 0.105 | 0.115 | 2.853 | 0.365 | 10 | 0.001 |
|  | Aldh5a1 | 0.786 | 1 | 0.786 | 1.147 | 0.296 | 0.050 | 1.147 | 0.176 | 24 | 0.002 |
|  | App | 0.097 | 1 | 0.097 | 0.144 | 0.708 | 0.007 | 0.144 | 0.065 | 53 | 0.004 |
|  | Bdnf | 2.320 | 1 | 2.320 | 2.647 | 0.118 | 0.107 | 2.647 | 0.343 | 11 | 0.001 |
|  | Cacna1a | 0.436 | 1 | 0.436 | 0.556 | 0.464 | 0.025 | 0.556 | 0.110 | 38 | 0.003 |
|  | Cdk5r1 | 0.775 | 1 | 0.775 | 1.227 | 0.280 | 0.053 | 1.227 | 0.185 | 23 | 0.002 |
|  | Dlg4 | 2.085 | 1 | 2.085 | 3.516 | 0.074 | 0.138 | 3.516 | 0.434 | 5 | 0.000 |
|  | Gabbr1 | 1.550 | 1 | 1.550 | 2.401 | 0.136 | 0.098 | 2.401 | 0.317 | 14 | 0.001 |
|  | Gabbr2 | 0.717 | 1 | 0.717 | 1.086 | 0.309 | 0.047 | 1.086 | 0.169 | 27 | 0.002 |
|  | Gabra1 | 1.141 | 1 | 1.141 | 1.116 | 0.302 | 0.048 | 1.116 | 0.173 | 25 | 0.002 |
|  | Gabra2 | 0.327 | 1 | 0.327 | 0.402 | 0.533 | 0.018 | 0.402 | 0.093 | 40 | 0.003 |
|  | Gabra4 | 0.685 | 1 | 0.685 | 1.007 | 0.326 | 0.044 | 1.007 | 0.161 | 29 | 0.002 |
|  | Gabra5 | 1.195 | 1 | 1.195 | 1.790 | 0.195 | 0.075 | 1.790 | 0.249 | 16 | 0.001 |
|  | Gabrb1 | 0.002 | 1 | 0.002 | 0.002 | 0.963 | 0.000 | 0.002 | 0.050 | 58 | 0.005 |
|  | Gabrb3 | 0.027 | 1 | 0.027 | 0.035 | 0.853 | 0.002 | 0.035 | 0.054 | 56 | 0.005 |
|  | Gabrd | 0.719 | 1 | 0.719 | 1.000 | 0.328 | 0.043 | 1.000 | 0.160 | 31 | 0.003 |
|  | Gabrg1 | 1.534 | 1 | 1.534 | 2.950 | 0.100 | 0.118 | 2.950 | 0.376 | 9 | 0.001 |
|  | Gabrg2 | 0.593 | 1 | 0.593 | 0.840 | 0.369 | 0.037 | 0.840 | 0.142 | 34 | 0.003 |
|  | Gad1 | 0.923 | 1 | 0.923 | 1.366 | 0.255 | 0.058 | 1.366 | 0.201 | 22 | 0.002 |
|  | Gls | 0.681 | 1 | 0.681 | 1.108 | 0.304 | 0.048 | 1.108 | 0.172 | 26 | 0.002 |
|  | Glul | 0.283 | 1 | 0.283 | 0.318 | 0.578 | 0.014 | 0.318 | 0.084 | 41 | 0.003 |
|  | Gnai1 | 0.000 | 1 | 0.000 | 0.001 | 0.982 | 0.000 | 0.001 | 0.050 | 60 | 0.005 |
|  | Gnaq | 0.385 | 1 | 0.385 | 0.607 | 0.445 | 0.028 | 0.607 | 0.115 | 37 | 0.003 |
|  | Gphn | 0.122 | 1 | 0.122 | 0.173 | 0.681 | 0.008 | 0.173 | 0.068 | 51 | 0.004 |
|  | Gria1 | 2.378 | 1 | 2.378 | 3.974 | 0.059 | 0.153 | 3.974 | 0.479 | 2 | 0.000 |
|  | Gria2 | 0.622 | 1 | 0.622 | 0.991 | 0.330 | 0.043 | 0.991 | 0.159 | 32 | 0.003 |
|  | Gria3 | 1.134 | 1 | 1.134 | 1.602 | 0.219 | 0.068 | 1.602 | 0.228 | 18 | 0.002 |
|  | Gria4 | 0.172 | 1 | 0.172 | 0.261 | 0.615 | 0.012 | 0.261 | 0.078 | 44 | 0.004 |
|  | Grik1 | 0.001 | 1 | 0.001 | 0.001 | 0.977 | 0.000 | 0.001 | 0.050 | 59 | 0.005 |
|  | Grik2 | 0.159 | 1 | 0.159 | 0.198 | 0.661 | 0.009 | 0.198 | 0.071 | 49 | 0.004 |
|  | Grik4 | 0.574 | 1 | 0.574 | 0.780 | 0.387 | 0.034 | 0.780 | 0.135 | 35 | 0.003 |
|  | Grik5 | 0.980 | 1 | 0.980 | 1.428 | 0.245 | 0.061 | 1.428 | 0.208 | 20 | 0.002 |
|  | Grin1 | 0.069 | 1 | 0.069 | 0.072 | 0.791 | 0.003 | 0.072 | 0.058 | 55 | 0.005 |
|  | Grin2a | 0.977 | 1 | 0.977 | 1.408 | 0.248 | 0.060 | 1.408 | 0.206 | 21 | 0.002 |
|  | Grin2b | 0.453 | 1 | 0.453 | 0.674 | 0.420 | 0.030 | 0.674 | 0.123 | 36 | 0.003 |
|  | Grm1 | 2.348 | 1 | 2.348 | 3.865 | 0.062 | 0.149 | 3.865 | 0.468 | 3 | 0.000 |
|  | Grm2 | 1.541 | 1 | 1.541 | 2.541 | 0.125 | 0.104 | 2.541 | 0.332 | 12 | 0.001 |
|  | Grm3 | 0.109 | 1 | 0.109 | 0.161 | 0.692 | 0.007 | 0.161 | 0.067 | 52 | 0.004 |
|  | Grm5 | 1.228 | 1 | 1.228 | 1.738 | 0.201 | 0.073 | 1.738 | 0.243 | 17 | 0.001 |
|  | Grm7 | 0.222 | 1 | 0.222 | 0.275 | 0.605 | 0.012 | 0.275 | 0.079 | 42 | 0.004 |
|  | Homer1 | 0.589 | 1 | 0.589 | 1.002 | 0.328 | 0.044 | 1.002 | 0.160 | 30 | 0.003 |
|  | Itpr1 | 0.049 | 1 | 0.049 | 0.072 | 0.790 | 0.003 | 0.072 | 0.058 | 54 | 0.005 |
|  | Mapk1 | 0.155 | 1 | 0.155 | 0.204 | 0.656 | 0.009 | 0.204 | 0.072 | 47 | 0.004 |
|  | Nsf | 2.064 | 1 | 2.064 | 3.612 | 0.071 | 0.141 | 3.612 | 0.443 | 4 | 0.000 |
|  | P2rx7 | 0.167 | 1 | 0.167 | 0.205 | 0.655 | 0.009 | 0.205 | 0.072 | 46 | 0.004 |
|  | Phgdh | 1.541 | 1 | 1.541 | 2.502 | 0.128 | 0.102 | 2.502 | 0.328 | 13 | 0.001 |
|  | Pla2g6 | 1.997 | 1 | 1.997 | 3.094 | 0.092 | 0.123 | 3.094 | 0.391 | 7 | 0.001 |
|  | Plcb1 | 0.566 | 1 | 0.566 | 0.872 | 0.360 | 0.038 | 0.872 | 0.145 | 33 | 0.003 |
|  | Prodh | 0.202 | 1 | 0.202 | 0.272 | 0.607 | 0.012 | 0.272 | 0.079 | 43 | 0.004 |
|  | Shank2 | 0.717 | 1 | 0.717 | 1.048 | 0.317 | 0.045 | 1.048 | 0.165 | 28 | 0.002 |
|  | Slc17a7 | 0.297 | 1 | 0.297 | 0.415 | 0.526 | 0.019 | 0.415 | 0.095 | 39 | 0.003 |
|  | Slc1a1 | 0.151 | 1 | 0.151 | 0.179 | 0.677 | 0.008 | 0.179 | 0.069 | 50 | 0.004 |
|  | Slc1a2 | 0.970 | 1 | 0.970 | 1.536 | 0.228 | 0.065 | 1.536 | 0.220 | 19 | 0.002 |
|  | Slc1a3 | 1.321 | 1 | 1.321 | 2.325 | 0.142 | 0.096 | 2.325 | 0.308 | 15 | 0.001 |
|  | Slc38a1 | 0.129 | 1 | 0.129 | 0.200 | 0.659 | 0.009 | 0.200 | 0.071 | 48 | 0.004 |
|  | Slc6a1 | 3.050 | 1 | 3.050 | 6.881 | 0.016 | 0.238 | 6.881 | 0.708 | 1 | 0.000 |
|  | Slc6a11 | 1.706 | 1 | 1.706 | 3.046 | 0.095 | 0.122 | 3.046 | 0.386 | 8 | 0.001 |
|  | Snca | 0.214 | 1 | 0.214 | 0.219 | 0.645 | 0.010 | 0.219 | 0.073 | 45 | 0.004 |
|  | Srr | 0.008 | 1 | 0.008 | 0.010 | 0.923 | 0.000 | 0.010 | 0.051 | 57 | 0.005 |
| Diet | Abat | 11.186 | 1 | 11.186 | 20.740 | 0.000 | 0.485 | 20.740 | 0.992 | 3 | 0.000 |
|  | Adora1 | 10.131 | 1 | 10.131 | 17.110 | 0.000 | 0.437 | 17.110 | 0.977 | 8 | 0.001 |
|  | Aldh5a1 | 8.641 | 1 | 8.641 | 12.613 | 0.002 | 0.364 | 12.613 | 0.924 | 31 | 0.003 |
|  | App | 8.696 | 1 | 8.696 | 12.924 | 0.002 | 0.370 | 12.924 | 0.930 | 28 | 0.002 |
|  | Bdnf | 2.316 | 1 | 2.316 | 2.643 | 0.118 | 0.107 | 2.643 | 0.343 | 57 | 0.005 |
|  | Cacna1a | 5.830 | 1 | 5.830 | 7.432 | 0.012 | 0.253 | 7.432 | 0.741 | 51 | 0.004 |
|  | Cdk5r1 | 9.919 | 1 | 9.919 | 15.704 | 0.001 | 0.417 | 15.704 | 0.966 | 12 | 0.001 |
|  | Dlg4 | 10.605 | 1 | 10.605 | 17.879 | 0.000 | 0.448 | 17.879 | 0.981 | 5 | 0.000 |
|  | Gabbr1 | 9.566 | 1 | 9.566 | 14.817 | 0.001 | 0.402 | 14.817 | 0.957 | 15 | 0.001 |
|  | Gabbr2 | 9.887 | 1 | 9.887 | 14.983 | 0.001 | 0.405 | 14.983 | 0.959 | 14 | 0.001 |
|  | Gabra1 | 0.722 | 1 | 0.722 | 0.707 | 0.410 | 0.031 | 0.707 | 0.127 | 60 | 0.005 |
|  | Gabra2 | 6.103 | 1 | 6.103 | 7.493 | 0.012 | 0.254 | 7.493 | 0.744 | 50 | 0.004 |
|  | Gabra4 | 8.793 | 1 | 8.793 | 12.925 | 0.002 | 0.370 | 12.925 | 0.930 | 27 | 0.002 |
|  | Gabra5 | 9.224 | 1 | 9.224 | 13.812 | 0.001 | 0.386 | 13.812 | 0.944 | 22 | 0.002 |
|  | Gabrb1 | 2.290 | 1 | 2.290 | 2.241 | 0.149 | 0.092 | 2.241 | 0.299 | 59 | 0.005 |
|  | Gabrb3 | 7.855 | 1 | 7.855 | 10.230 | 0.004 | 0.317 | 10.230 | 0.863 | 42 | 0.004 |
|  | Gabrd | 6.913 | 1 | 6.913 | 9.619 | 0.005 | 0.304 | 9.619 | 0.842 | 45 | 0.004 |
|  | Gabrg1 | 11.916 | 1 | 11.916 | 22.921 | 0.000 | 0.510 | 22.921 | 0.995 | 1 | 0.000 |
|  | Gabrg2 | 8.211 | 1 | 8.211 | 11.634 | 0.003 | 0.346 | 11.634 | 0.903 | 38 | 0.003 |
|  | Gad1 | 8.136 | 1 | 8.136 | 12.039 | 0.002 | 0.354 | 12.039 | 0.912 | 37 | 0.003 |
|  | Gls | 8.400 | 1 | 8.400 | 13.678 | 0.001 | 0.383 | 13.678 | 0.942 | 23 | 0.002 |
|  | Glul | 2.550 | 1 | 2.550 | 2.867 | 0.105 | 0.115 | 2.867 | 0.367 | 56 | 0.005 |
|  | Gnai1 | 8.693 | 1 | 8.693 | 12.144 | 0.002 | 0.356 | 12.144 | 0.915 | 36 | 0.003 |
|  | Gnaq | 10.223 | 1 | 10.223 | 16.126 | 0.001 | 0.434 | 16.126 | 0.969 | 11 | 0.001 |
|  | Gphn | 8.735 | 1 | 8.735 | 12.423 | 0.002 | 0.361 | 12.423 | 0.920 | 34 | 0.003 |
|  | Gria1 | 8.562 | 1 | 8.562 | 14.309 | 0.001 | 0.394 | 14.309 | 0.951 | 18 | 0.002 |
|  | Gria2 | 7.927 | 1 | 7.927 | 12.626 | 0.002 | 0.365 | 12.626 | 0.924 | 30 | 0.003 |
|  | Gria3 | 7.511 | 1 | 7.511 | 10.613 | 0.004 | 0.325 | 10.613 | 0.875 | 41 | 0.003 |
|  | Gria4 | 8.316 | 1 | 8.316 | 12.596 | 0.002 | 0.364 | 12.596 | 0.924 | 32 | 0.003 |
|  | Grik1 | 6.210 | 1 | 6.210 | 7.797 | 0.011 | 0.262 | 7.797 | 0.761 | 49 | 0.004 |
|  | Grik2 | 6.889 | 1 | 6.889 | 8.589 | 0.008 | 0.281 | 8.589 | 0.800 | 48 | 0.004 |
|  | Grik4 | 7.302 | 1 | 7.302 | 9.918 | 0.005 | 0.311 | 9.918 | 0.853 | 44 | 0.004 |
|  | Grik5 | 7.837 | 1 | 7.837 | 11.418 | 0.003 | 0.342 | 11.418 | 0.898 | 40 | 0.003 |
|  | Grin1 | 2.335 | 1 | 2.335 | 2.430 | 0.133 | 0.099 | 2.430 | 0.320 | 58 | 0.005 |
|  | Grin2a | 8.734 | 1 | 8.734 | 12.584 | 0.002 | 0.364 | 12.584 | 0.923 | 33 | 0.003 |
|  | Grin2b | 9.317 | 1 | 9.317 | 13.879 | 0.001 | 0.387 | 13.879 | 0.945 | 21 | 0.002 |
|  | Grm1 | 8.735 | 1 | 8.735 | 14.380 | 0.001 | 0.395 | 14.380 | 0.952 | 16 | 0.001 |
|  | Grm2 | 8.694 | 1 | 8.694 | 14.337 | 0.001 | 0.395 | 14.337 | 0.951 | 17 | 0.001 |
|  | Grm3 | 9.648 | 1 | 9.648 | 14.264 | 0.001 | 0.393 | 14.264 | 0.950 | 19 | 0.002 |
|  | Grm5 | 7.103 | 1 | 7.103 | 10.055 | 0.004 | 0.314 | 10.055 | 0.858 | 43 | 0.004 |
|  | Grm7 | 6.950 | 1 | 6.950 | 8.627 | 0.008 | 0.282 | 8.627 | 0.801 | 47 | 0.004 |
|  | Homer1 | 10.102 | 1 | 10.102 | 17.184 | 0.000 | 0.439 | 17.184 | 0.977 | 7 | 0.001 |
|  | Itpr1 | 9.504 | 1 | 9.504 | 14.100 | 0.001 | 0.391 | 14.100 | 0.948 | 20 | 0.002 |
|  | Mapk1 | 5.677 | 1 | 5.677 | 7.432 | 0.012 | 0.253 | 7.432 | 0.741 | 52 | 0.004 |
|  | Nsf | 9.148 | 1 | 9.148 | 16.013 | 0.001 | 0.421 | 16.013 | 0.969 | 10 | 0.001 |
|  | P2rx7 | 5.560 | 1 | 5.560 | 6.794 | 0.016 | 0.236 | 6.794 | 0.702 | 53 | 0.004 |
|  | Phgdh | 8.326 | 1 | 8.326 | 13.515 | 0.001 | 0.381 | 13.515 | 0.940 | 25 | 0.002 |
|  | Pla2g6 | 8.723 | 1 | 8.723 | 13.518 | 0.001 | 0.381 | 13.518 | 0.940 | 24 | 0.002 |
|  | Plcb1 | 8.406 | 1 | 8.406 | 12.963 | 0.002 | 0.371 | 12.963 | 0.930 | 26 | 0.002 |
|  | Prodh | 8.626 | 1 | 8.626 | 11.602 | 0.003 | 0.345 | 11.602 | 0.902 | 39 | 0.003 |
|  | Shank2 | 8.825 | 1 | 8.825 | 12.904 | 0.002 | 0.370 | 12.904 | 0.929 | 29 | 0.002 |
|  | Slc17a7 | 8.684 | 1 | 8.684 | 12.153 | 0.002 | 0.356 | 12.153 | 0.915 | 35 | 0.003 |
|  | Slc1a1 | 5.611 | 1 | 5.611 | 6.619 | 0.017 | 0.231 | 6.619 | 0.691 | 54 | 0.005 |
|  | Slc1a2 | 10.617 | 1 | 10.617 | 16.819 | 0.000 | 0.433 | 16.819 | 0.975 | 9 | 0.001 |
|  | Slc1a3 | 9.930 | 1 | 9.930 | 17.475 | 0.000 | 0.443 | 17.475 | 0.979 | 6 | 0.001 |
|  | Slc38a1 | 9.713 | 1 | 9.713 | 15.105 | 0.001 | 0.407 | 15.105 | 0.960 | 13 | 0.001 |
|  | Slc6a1 | 9.924 | 1 | 9.924 | 22.387 | 0.000 | 0.504 | 22.387 | 0.995 | 2 | 0.000 |
|  | Slc6a11 | 11.102 | 1 | 11.102 | 19.820 | 0.000 | 0.474 | 19.820 | 0.989 | 4 | 0.000 |
|  | Snca | 3.315 | 1 | 3.315 | 3.386 | 0.079 | 0.133 | 3.386 | 0.421 | 55 | 0.005 |
|  | Srr | 6.938 | 1 | 6.938 | 8.753 | 0.007 | 0.285 | 8.753 | 0.807 | 46 | 0.004 |
| Age * Diet | Abat | 0.346 | 1 | 0.346 | 0.642 | 0.431 | 0.028 | 0.642 | 0.120 | 25 | 0.002 |
|  | Adora1 | 0.337 | 1 | 0.337 | 0.570 | 0.458 | 0.025 | 0.570 | 0.112 | 28 | 0.002 |
|  | Aldh5a1 | 0.366 | 1 | 0.366 | 0.534 | 0.473 | 0.024 | 0.534 | 0.108 | 31 | 0.003 |
|  | App | 0.663 | 1 | 0.663 | 0.986 | 0.332 | 0.043 | 0.986 | 0.158 | 21 | 0.002 |
|  | Bdnf | 0.034 | 1 | 0.034 | 0.039 | 0.845 | 0.002 | 0.039 | 0.054 | 55 | 0.005 |
|  | Cacna1a | 0.949 | 1 | 0.949 | 1.210 | 0.283 | 0.052 | 1.210 | 0.183 | 15 | 0.001 |
|  | Cdk5r1 | 0.304 | 1 | 0.304 | 0.482 | 0.495 | 0.021 | 0.482 | 0.102 | 34 | 0.003 |
|  | Dlg4 | 0.039 | 1 | 0.039 | 0.066 | 0.799 | 0.003 | 0.066 | 0.057 | 52 | 0.004 |
|  | Gabbr1 | 0.110 | 1 | 0.110 | 0.171 | 0.683 | 0.008 | 0.171 | 0.068 | 45 | 0.004 |
|  | Gabbr2 | 0.060 | 1 | 0.060 | 0.090 | 0.767 | 0.004 | 0.090 | 0.060 | 49 | 0.004 |
|  | Gabra1 | 0.138 | 1 | 0.138 | 0.135 | 0.717 | 0.006 | 0.135 | 0.064 | 46 | 0.004 |
|  | Gabra2 | 0.371 | 1 | 0.371 | 0.455 | 0.507 | 0.020 | 0.455 | 0.099 | 35 | 0.003 |
|  | Gabra4 | 0.371 | 1 | 0.371 | 0.546 | 0.468 | 0.024 | 0.546 | 0.109 | 30 | 0.003 |
|  | Gabra5 | 0.143 | 1 | 0.143 | 0.213 | 0.649 | 0.010 | 0.213 | 0.073 | 42 | 0.004 |
|  | Gabrb1 | 0.319 | 1 | 0.319 | 0.312 | 0.582 | 0.014 | 0.312 | 0.083 | 40 | 0.003 |
|  | Gabrb3 | 0.000 | 1 | 0.000 | 0.000 | 0.991 | 0.000 | 0.000 | 0.050 | 60 | 0.005 |
|  | Gabrd | 1.033 | 1 | 1.033 | 1.437 | 0.243 | 0.061 | 1.437 | 0.209 | 12 | 0.001 |
|  | Gabrg1 | 0.296 | 1 | 0.296 | 0.569 | 0.459 | 0.025 | 0.569 | 0.112 | 29 | 0.002 |
|  | Gabrg2 | 0.419 | 1 | 0.419 | 0.594 | 0.449 | 0.026 | 0.594 | 0.114 | 27 | 0.002 |
|  | Gad1 | 0.735 | 1 | 0.735 | 1.088 | 0.308 | 0.047 | 1.088 | 0.170 | 18 | 0.002 |
|  | Gls | 1.577 | 1 | 1.577 | 2.567 | 0.123 | 0.105 | 2.567 | 0.335 | 4 | 0.000 |
|  | Glul | 2.285 | 1 | 2.285 | 2.569 | 0.123 | 0.105 | 2.569 | 0.335 | 3 | 0.000 |
|  | Gnai1 | 0.036 | 1 | 0.036 | 0.050 | 0.825 | 0.002 | 0.050 | 0.055 | 54 | 0.005 |
|  | Gnaq | 0.009 | 1 | 0.009 | 0.014 | 0.908 | 0.001 | 0.014 | 0.051 | 56 | 0.005 |
|  | Gphn | 0.232 | 1 | 0.232 | 0.329 | 0.572 | 0.015 | 0.329 | 0.085 | 39 | 0.003 |
|  | Gria1 | 0.820 | 1 | 0.820 | 1.371 | 0.254 | 0.059 | 1.371 | 0.202 | 14 | 0.001 |
|  | Gria2 | 1.746 | 1 | 1.746 | 2.781 | 0.110 | 0.112 | 2.781 | 0.358 | 2 | 0.000 |
|  | Gria3 | 0.602 | 1 | 0.602 | 0.850 | 0.367 | 0.037 | 0.850 | 0.143 | 22 | 0.002 |
|  | Gria4 | 1.128 | 1 | 1.128 | 1.708 | 0.205 | 0.072 | 1.708 | 0.240 | 9 | 0.001 |
|  | Grik1 | 0.556 | 1 | 0.556 | 0.698 | 0.412 | 0.031 | 0.698 | 0.126 | 24 | 0.002 |
|  | Grik2 | 0.104 | 1 | 0.104 | 0.130 | 0.722 | 0.006 | 0.130 | 0.064 | 47 | 0.004 |
|  | Grik4 | 0.583 | 1 | 0.583 | 0.791 | 0.383 | 0.035 | 0.791 | 0.136 | 23 | 0.002 |
|  | Grik5 | 0.757 | 1 | 0.757 | 1.103 | 0.305 | 0.048 | 1.103 | 0.171 | 17 | 0.001 |
|  | Grin1 | 1.003 | 1 | 1.003 | 1.044 | 0.318 | 0.045 | 1.044 | 0.165 | 19 | 0.002 |
|  | Grin2a | 0.164 | 1 | 0.164 | 0.236 | 0.632 | 0.011 | 0.236 | 0.075 | 41 | 0.003 |
|  | Grin2b | 0.251 | 1 | 0.251 | 0.374 | 0.547 | 0.017 | 0.374 | 0.090 | 38 | 0.003 |
|  | Grm1 | 0.621 | 1 | 0.621 | 1.022 | 0.323 | 0.044 | 1.022 | 0.162 | 20 | 0.002 |
|  | Grm2 | 1.049 | 1 | 1.049 | 1.731 | 0.202 | 0.073 | 1.731 | 0.242 | 7 | 0.001 |
|  | Grm3 | 0.060 | 1 | 0.060 | 0.088 | 0.769 | 0.004 | 0.088 | 0.059 | 50 | 0.004 |
|  | Grm5 | 0.830 | 1 | 0.830 | 1.174 | 0.290 | 0.051 | 1.174 | 0.179 | 16 | 0.001 |
|  | Grm7 | 0.043 | 1 | 0.043 | 0.053 | 0.820 | 0.002 | 0.053 | 0.056 | 53 | 0.004 |
|  | Homer1 | 0.813 | 1 | 0.813 | 1.383 | 0.252 | 0.059 | 1.383 | 0.203 | 13 | 0.001 |
|  | Itpr1 | 0.127 | 1 | 0.127 | 0.189 | 0.668 | 0.008 | 0.189 | 0.070 | 43 | 0.004 |
|  | Mapk1 | 1.528 | 1 | 1.528 | 2.001 | 0.171 | 0.083 | 2.001 | 0.272 | 5 | 0.000 |
|  | Nsf | 0.984 | 1 | 0.984 | 1.723 | 0.203 | 0.073 | 1.723 | 0.241 | 8 | 0.001 |
|  | P2rx7 | 0.397 | 1 | 0.397 | 0.485 | 0.493 | 0.022 | 0.485 | 0.102 | 33 | 0.003 |
|  | Phgdh | 1.150 | 1 | 1.150 | 1.867 | 0.186 | 0.078 | 1.867 | 0.257 | 6 | 0.001 |
|  | Pla2g6 | 0.336 | 1 | 0.336 | 0.521 | 0.478 | 0.023 | 0.521 | 0.106 | 32 | 0.003 |
|  | Plcb1 | 1.103 | 1 | 1.103 | 1.701 | 0.206 | 0.072 | 1.701 | 0.239 | 10 | 0.001 |
|  | Prodh | 0.095 | 1 | 0.095 | 0.128 | 0.724 | 0.006 | 0.128 | 0.064 | 48 | 0.004 |
|  | Shank2 | 0.301 | 1 | 0.301 | 0.441 | 0.514 | 0.020 | 0.441 | 0.097 | 36 | 0.003 |
|  | Slc17a7 | 0.128 | 1 | 0.128 | 0.179 | 0.676 | 0.008 | 0.179 | 0.069 | 44 | 0.004 |
|  | Slc1a1 | 0.005 | 1 | 0.005 | 0.006 | 0.941 | 0.000 | 0.006 | 0.051 | 59 | 0.005 |
|  | Slc1a2 | 0.006 | 1 | 0.006 | 0.010 | 0.921 | 0.000 | 0.010 | 0.051 | 57 | 0.005 |
|  | Slc1a3 | 0.894 | 1 | 0.894 | 1.574 | 0.223 | 0.067 | 1.574 | 0.224 | 11 | 0.001 |
|  | Slc38a1 | 0.404 | 1 | 0.404 | 0.628 | 0.436 | 0.028 | 0.628 | 0.118 | 26 | 0.002 |
|  | Slc6a1 | 1.744 | 1 | 1.744 | 3.934 | 0.060 | 0.152 | 3.934 | 0.475 | 1 | 0.000 |
|  | Slc6a11 | 0.211 | 1 | 0.211 | 0.377 | 0.545 | 0.017 | 0.377 | 0.090 | 37 | 0.003 |
|  | Snca | 0.009 | 1 | 0.009 | 0.009 | 0.925 | 0.000 | 0.009 | 0.051 | 58 | 0.005 |
|  | Srr | 0.061 | 1 | 0.061 | 0.077 | 0.783 | 0.004 | 0.077 | 0.058 | 51 | 0.004 |

Table S4: Statisical summary for DG gene expression.

| Gene | Age | Diet | Mean (Z-Scores) | Std. Deviation | N |
| --- | --- | --- | --- | --- | --- |
| Abat | Young | Standard | 0.794 | 0.779 | 7 |
|  |  | Ketogenic | -0.085 | 0.832 | 6 |
|  |  | Total | 0.388 | 0.894 | 13 |
|  | Aged | Standard | 0.159 | 0.663 | 8 |
|  |  | Ketogenic | -1.053 | 0.950 | 6 |
|  |  | Total | -0.360 | 0.986 | 14 |
|  | Total | Standard | 0.455 | 0.766 | 15 |
|  |  | Ketogenic | -0.569 | 0.990 | 12 |
|  |  | Total | 0.000 | 1.000 | 27 |
| Adora1 | Young | Standard | 0.540 | 1.065 | 7 |
|  |  | Ketogenic | 0.328 | 0.905 | 6 |
|  |  | Total | 0.442 | 0.960 | 13 |
|  | Aged | Standard | 0.119 | 0.612 | 8 |
|  |  | Ketogenic | -1.116 | 0.663 | 6 |
|  |  | Total | -0.410 | 0.879 | 14 |
|  | Total | Standard | 0.316 | 0.849 | 15 |
|  |  | Ketogenic | -0.394 | 1.068 | 12 |
|  |  | Total | 0.000 | 1.000 | 27 |
| Aldh5a1 | Young | Standard | 0.721 | 0.759 | 7 |
|  |  | Ketogenic | 0.098 | 0.870 | 6 |
|  |  | Total | 0.434 | 0.841 | 13 |
|  | Aged | Standard | 0.177 | 0.574 | 8 |
|  |  | Ketogenic | -1.175 | 0.918 | 6 |
|  |  | Total | -0.403 | 0.992 | 14 |
|  | Total | Standard | 0.431 | 0.700 | 15 |
|  |  | Ketogenic | -0.539 | 1.081 | 12 |
|  |  | Total | 0.000 | 1.000 | 27 |
| App | Young | Standard | 0.852 | 0.499 | 7 |
|  |  | Ketogenic | 0.317 | 0.523 | 6 |
|  |  | Total | 0.605 | 0.562 | 13 |
|  | Aged | Standard | -0.098 | 0.319 | 8 |
|  |  | Ketogenic | -1.180 | 1.289 | 6 |
|  |  | Total | -0.562 | 1.001 | 14 |
|  | Total | Standard | 0.345 | 0.631 | 15 |
|  |  | Ketogenic | -0.432 | 1.221 | 12 |
|  |  | Total | 0.000 | 1.000 | 27 |
| Bdnf | Young | Standard | 0.305 | 0.751 | 7 |
|  |  | Ketogenic | 0.561 | 1.047 | 6 |
|  |  | Total | 0.423 | 0.870 | 13 |
|  | Aged | Standard | -0.054 | 0.737 | 8 |
|  |  | Ketogenic | -1.543 | 1.293 | 5 |
|  |  | Total | -0.627 | 1.201 | 13 |
|  | Total | Standard | 0.113 | 0.740 | 15 |
|  |  | Ketogenic | -0.395 | 1.557 | 11 |
|  |  | Total | -0.102 | 1.158 | 26 |
| Cacna1a | Young | Standard | 0.549 | 1.153 | 7 |
|  |  | Ketogenic | 0.346 | 1.084 | 6 |
|  |  | Total | 0.455 | 1.080 | 13 |
|  | Aged | Standard | 0.025 | 0.500 | 8 |
|  |  | Ketogenic | -1.020 | 0.515 | 6 |
|  |  | Total | -0.423 | 0.724 | 14 |
|  | Total | Standard | 0.270 | 0.877 | 15 |
|  |  | Ketogenic | -0.337 | 1.078 | 12 |
|  |  | Total | 0.000 | 1.000 | 27 |
| Cdk5r1 | Young | Standard | 0.682 | 0.960 | 7 |
|  |  | Ketogenic | 0.325 | 0.794 | 6 |
|  |  | Total | 0.517 | 0.870 | 13 |
|  | Aged | Standard | 0.053 | 0.531 | 8 |
|  |  | Ketogenic | -1.190 | 0.766 | 6 |
|  |  | Total | -0.480 | 0.886 | 14 |
|  | Total | Standard | 0.346 | 0.801 | 15 |
|  |  | Ketogenic | -0.433 | 1.086 | 12 |
|  |  | Total | 0.000 | 1.000 | 27 |
| Dlg4 | Young | Standard | 0.647 | 0.640 | 7 |
|  |  | Ketogenic | 0.085 | 0.892 | 6 |
|  |  | Total | 0.388 | 0.788 | 13 |
|  | Aged | Standard | 0.240 | 0.686 | 8 |
|  |  | Ketogenic | -1.159 | 0.977 | 6 |
|  |  | Total | -0.360 | 1.066 | 14 |
|  | Total | Standard | 0.430 | 0.675 | 15 |
|  |  | Ketogenic | -0.537 | 1.103 | 12 |
|  |  | Total | 0.000 | 1.000 | 27 |
| Gabbr1 | Young | Standard | 0.515 | 0.998 | 7 |
|  |  | Ketogenic | 0.069 | 0.959 | 6 |
|  |  | Total | 0.309 | 0.967 | 13 |
|  | Aged | Standard | 0.200 | 0.723 | 8 |
|  |  | Ketogenic | -0.936 | 0.927 | 6 |
|  |  | Total | -0.287 | 0.976 | 14 |
|  | Total | Standard | 0.347 | 0.846 | 15 |
|  |  | Ketogenic | -0.434 | 1.041 | 12 |
|  |  | Total | 0.000 | 1.000 | 27 |
| Gabbr2 | Young | Standard | 0.751 | 0.766 | 7 |
|  |  | Ketogenic | 0.314 | 0.843 | 6 |
|  |  | Total | 0.549 | 0.801 | 13 |
|  | Aged | Standard | -0.043 | 0.559 | 8 |
|  |  | Ketogenic | -1.132 | 0.950 | 6 |
|  |  | Total | -0.510 | 0.910 | 14 |
|  | Total | Standard | 0.327 | 0.759 | 15 |
|  |  | Ketogenic | -0.409 | 1.141 | 12 |
|  |  | Total | 0.000 | 1.000 | 27 |
| Gabra1 | Young | Standard | 0.240 | 0.436 | 7 |
|  |  | Ketogenic | 0.655 | 0.928 | 6 |
|  |  | Total | 0.431 | 0.707 | 13 |
|  | Aged | Standard | -0.165 | 0.918 | 8 |
|  |  | Ketogenic | -0.714 | 1.296 | 6 |
|  |  | Total | -0.400 | 1.086 | 14 |
|  | Total | Standard | 0.024 | 0.740 | 15 |
|  |  | Ketogenic | -0.030 | 1.291 | 12 |
|  |  | Total | 0.000 | 1.000 | 27 |
| Gabra2 | Young | Standard | 0.623 | 0.434 | 7 |
|  |  | Ketogenic | 0.488 | 0.679 | 6 |
|  |  | Total | 0.561 | 0.540 | 13 |
|  | Aged | Standard | -0.284 | 1.017 | 8 |
|  |  | Ketogenic | -0.835 | 1.127 | 6 |
|  |  | Total | -0.520 | 1.061 | 14 |
|  | Total | Standard | 0.139 | 0.904 | 15 |
|  |  | Ketogenic | -0.174 | 1.124 | 12 |
|  |  | Total | 0.000 | 1.000 | 27 |
| Gabra4 | Young | Standard | 0.662 | 0.686 | 7 |
|  |  | Ketogenic | 0.141 | 0.601 | 6 |
|  |  | Total | 0.421 | 0.678 | 13 |
|  | Aged | Standard | 0.091 | 1.129 | 8 |
|  |  | Ketogenic | -1.035 | 0.744 | 6 |
|  |  | Total | -0.391 | 1.111 | 14 |
|  | Total | Standard | 0.357 | 0.962 | 15 |
|  |  | Ketogenic | -0.447 | 0.891 | 12 |
|  |  | Total | 0.000 | 1.000 | 27 |
| Gabra5 | Young | Standard | 0.835 | 0.547 | 7 |
|  |  | Ketogenic | 0.299 | 0.766 | 6 |
|  |  | Total | 0.587 | 0.686 | 13 |
|  | Aged | Standard | -0.106 | 0.490 | 8 |
|  |  | Ketogenic | -1.131 | 1.133 | 6 |
|  |  | Total | -0.545 | 0.949 | 14 |
|  | Total | Standard | 0.333 | 0.696 | 15 |
|  |  | Ketogenic | -0.416 | 1.186 | 12 |
|  |  | Total | 0.000 | 1.000 | 27 |
| Gabrb1 | Young | Standard | 0.159 | 0.499 | 7 |
|  |  | Ketogenic | 0.863 | 0.834 | 6 |
|  |  | Total | 0.484 | 0.740 | 13 |
|  | Aged | Standard | -0.119 | 0.914 | 8 |
|  |  | Ketogenic | -0.891 | 1.065 | 6 |
|  |  | Total | -0.450 | 1.021 | 14 |
|  | Total | Standard | 0.011 | 0.738 | 15 |
|  |  | Ketogenic | -0.014 | 1.292 | 12 |
|  |  | Total | 0.000 | 1.000 | 27 |
| Gabrb3 | Young | Standard | 0.452 | 0.652 | 7 |
|  |  | Ketogenic | 0.649 | 0.909 | 6 |
|  |  | Total | 0.543 | 0.753 | 13 |
|  | Aged | Standard | -0.112 | 0.805 | 8 |
|  |  | Ketogenic | -1.026 | 0.939 | 6 |
|  |  | Total | -0.504 | 0.953 | 14 |
|  | Total | Standard | 0.151 | 0.769 | 15 |
|  |  | Ketogenic | -0.189 | 1.242 | 12 |
|  |  | Total | 0.000 | 1.000 | 27 |
| Gabrg1 | Young | Standard | 0.760 | 0.379 | 7 |
|  |  | Ketogenic | -0.016 | 0.435 | 6 |
|  |  | Total | 0.402 | 0.559 | 13 |
|  | Aged | Standard | -0.078 | 1.162 | 8 |
|  |  | Ketogenic | -0.766 | 1.193 | 6 |
|  |  | Total | -0.373 | 1.183 | 14 |
|  | Total | Standard | 0.313 | 0.961 | 15 |
|  |  | Ketogenic | -0.391 | 0.942 | 12 |
|  |  | Total | 0.000 | 1.000 | 27 |
| Gabrg2 | Young | Standard | 0.750 | 0.412 | 7 |
|  |  | Ketogenic | 0.318 | 0.414 | 6 |
|  |  | Total | 0.550 | 0.454 | 13 |
|  | Aged | Standard | -0.242 | 1.106 | 8 |
|  |  | Ketogenic | -0.870 | 1.096 | 6 |
|  |  | Total | -0.511 | 1.107 | 14 |
|  | Total | Standard | 0.221 | 0.973 | 15 |
|  |  | Ketogenic | -0.276 | 1.005 | 12 |
|  |  | Total | 0.000 | 1.000 | 27 |
| Gabrg3 | Young | Standard | 0.209 | 0.894 | 7 |
|  |  | Ketogenic | 0.942 | 0.745 | 6 |
|  |  | Total | 0.547 | 0.881 | 13 |
|  | Aged | Standard | -1.218 | 1.404 | 8 |
|  |  | Ketogenic | -1.813 | 2.248 | 6 |
|  |  | Total | -1.473 | 1.760 | 14 |
|  | Total | Standard | -0.552 | 1.368 | 15 |
|  |  | Ketogenic | -0.436 | 2.149 | 12 |
|  |  | Total | -0.500 | 1.722 | 27 |
| Gad1 | Young | Standard | 0.764 | 0.868 | 7 |
|  |  | Ketogenic | 0.294 | 0.806 | 6 |
|  |  | Total | 0.547 | 0.841 | 13 |
|  | Aged | Standard | -0.349 | 0.548 | 7 |
|  |  | Ketogenic | -0.778 | 1.126 | 6 |
|  |  | Total | -0.547 | 0.853 | 13 |
|  | Total | Standard | 0.208 | 0.906 | 14 |
|  |  | Ketogenic | -0.242 | 1.088 | 12 |
|  |  | Total | 0.000 | 1.000 | 26 |
| Gls | Young | Standard | 0.999 | 0.828 | 7 |
|  |  | Ketogenic | 0.168 | 0.693 | 6 |
|  |  | Total | 0.615 | 0.854 | 13 |
|  | Aged | Standard | -0.283 | 0.670 | 8 |
|  |  | Ketogenic | -0.956 | 0.782 | 6 |
|  |  | Total | -0.571 | 0.773 | 14 |
|  | Total | Standard | 0.315 | 0.978 | 15 |
|  |  | Ketogenic | -0.394 | 0.917 | 12 |
|  |  | Total | 0.000 | 1.000 | 27 |
| Glul | Young | Standard | -0.296 | 0.745 | 7 |
|  |  | Ketogenic | 0.724 | 0.881 | 6 |
|  |  | Total | 0.174 | 0.938 | 13 |
|  | Aged | Standard | 0.404 | 0.713 | 8 |
|  |  | Ketogenic | -0.917 | 1.013 | 6 |
|  |  | Total | -0.162 | 1.062 | 14 |
|  | Total | Standard | 0.077 | 0.789 | 15 |
|  |  | Ketogenic | -0.097 | 1.246 | 12 |
|  |  | Total | 0.000 | 1.000 | 27 |
| Gnai1 | Young | Standard | 0.725 | 0.606 | 7 |
|  |  | Ketogenic | 0.476 | 0.891 | 6 |
|  |  | Total | 0.610 | 0.729 | 13 |
|  | Aged | Standard | -0.341 | 0.630 | 8 |
|  |  | Ketogenic | -0.868 | 1.145 | 6 |
|  |  | Total | -0.567 | 0.890 | 14 |
|  | Total | Standard | 0.157 | 0.812 | 15 |
|  |  | Ketogenic | -0.196 | 1.204 | 12 |
|  |  | Total | 0.000 | 1.000 | 27 |
| Gnaq | Young | Standard | 0.884 | 0.747 | 7 |
|  |  | Ketogenic | 0.267 | 0.816 | 6 |
|  |  | Total | 0.599 | 0.812 | 13 |
|  | Aged | Standard | -0.146 | 0.522 | 8 |
|  |  | Ketogenic | -1.104 | 0.898 | 6 |
|  |  | Total | -0.557 | 0.836 | 14 |
|  | Total | Standard | 0.335 | 0.811 | 15 |
|  |  | Ketogenic | -0.418 | 1.087 | 12 |
|  |  | Total | 0.000 | 1.000 | 27 |
| Gphn | Young | Standard | 0.844 | 0.959 | 7 |
|  |  | Ketogenic | 0.278 | 0.717 | 6 |
|  |  | Total | 0.582 | 0.872 | 13 |
|  | Aged | Standard | -0.188 | 0.519 | 8 |
|  |  | Ketogenic | -1.011 | 0.912 | 6 |
|  |  | Total | -0.541 | 0.802 | 14 |
|  | Total | Standard | 0.293 | 0.902 | 15 |
|  |  | Ketogenic | -0.367 | 1.032 | 12 |
|  |  | Total | 0.000 | 1.000 | 27 |
| Gria1 | Young | Standard | 0.763 | 0.482 | 7 |
|  |  | Ketogenic | 0.115 | 0.679 | 6 |
|  |  | Total | 0.464 | 0.649 | 13 |
|  | Aged | Standard | 0.026 | 0.776 | 8 |
|  |  | Ketogenic | -1.039 | 1.220 | 6 |
|  |  | Total | -0.431 | 1.094 | 14 |
|  | Total | Standard | 0.370 | 0.739 | 15 |
|  |  | Ketogenic | -0.462 | 1.118 | 12 |
|  |  | Total | 0.000 | 1.000 | 27 |
| Gria2 | Young | Standard | 0.656 | 0.446 | 7 |
|  |  | Ketogenic | 0.150 | 0.505 | 6 |
|  |  | Total | 0.423 | 0.524 | 13 |
|  | Aged | Standard | -0.117 | 1.251 | 8 |
|  |  | Ketogenic | -0.761 | 1.081 | 6 |
|  |  | Total | -0.393 | 1.184 | 14 |
|  | Total | Standard | 0.244 | 1.014 | 15 |
|  |  | Ketogenic | -0.305 | 0.935 | 12 |
|  |  | Total | 0.000 | 1.000 | 27 |
| Gria3 | Young | Standard | 0.728 | 0.470 | 7 |
|  |  | Ketogenic | 0.152 | 0.397 | 6 |
|  |  | Total | 0.462 | 0.515 | 13 |
|  | Aged | Standard | -0.080 | 1.161 | 8 |
|  |  | Ketogenic | -0.895 | 1.072 | 6 |
|  |  | Total | -0.429 | 1.159 | 14 |
|  | Total | Standard | 0.297 | 0.971 | 15 |
|  |  | Ketogenic | -0.371 | 0.945 | 12 |
|  |  | Total | 0.000 | 1.000 | 27 |
| Gria4 | Young | Standard | 0.777 | 0.545 | 7 |
|  |  | Ketogenic | 0.074 | 0.645 | 6 |
|  |  | Total | 0.452 | 0.674 | 13 |
|  | Aged | Standard | -0.124 | 0.948 | 8 |
|  |  | Ketogenic | -0.815 | 1.224 | 6 |
|  |  | Total | -0.420 | 1.089 | 14 |
|  | Total | Standard | 0.296 | 0.890 | 15 |
|  |  | Ketogenic | -0.370 | 1.042 | 12 |
|  |  | Total | 0.000 | 1.000 | 27 |
| Grik2 | Young | Standard | 0.698 | 0.471 | 7 |
|  |  | Ketogenic | 0.466 | 0.698 | 6 |
|  |  | Total | 0.591 | 0.573 | 13 |
|  | Aged | Standard | -0.273 | 0.899 | 8 |
|  |  | Ketogenic | -0.917 | 1.115 | 6 |
|  |  | Total | -0.549 | 1.011 | 14 |
|  | Total | Standard | 0.180 | 0.867 | 15 |
|  |  | Ketogenic | -0.225 | 1.144 | 12 |
|  |  | Total | 0.000 | 1.000 | 27 |
| Grik4 | Young | Standard | 1.005 | 0.971 | 7 |
|  |  | Ketogenic | 0.161 | 0.791 | 6 |
|  |  | Total | 0.616 | 0.961 | 13 |
|  | Aged | Standard | -0.203 | 0.359 | 8 |
|  |  | Ketogenic | -1.064 | 0.625 | 6 |
|  |  | Total | -0.572 | 0.644 | 14 |
|  | Total | Standard | 0.361 | 0.926 | 15 |
|  |  | Ketogenic | -0.451 | 0.934 | 12 |
|  |  | Total | 0.000 | 1.000 | 27 |
| Grik5 | Young | Standard | 0.751 | 0.961 | 7 |
|  |  | Ketogenic | 0.200 | 1.032 | 6 |
|  |  | Total | 0.497 | 0.994 | 13 |
|  | Aged | Standard | -0.044 | 0.627 | 8 |
|  |  | Ketogenic | -1.018 | 0.626 | 6 |
|  |  | Total | -0.461 | 0.783 | 14 |
|  | Total | Standard | 0.327 | 0.872 | 15 |
|  |  | Ketogenic | -0.409 | 1.033 | 12 |
|  |  | Total | 0.000 | 1.000 | 27 |
| Grin1 | Young | Standard | 0.776 | 0.586 | 7 |
|  |  | Ketogenic | 0.495 | 0.810 | 6 |
|  |  | Total | 0.647 | 0.683 | 13 |
|  | Aged | Standard | -0.204 | 0.763 | 8 |
|  |  | Ketogenic | -1.129 | 0.764 | 6 |
|  |  | Total | -0.600 | 0.874 | 14 |
|  | Total | Standard | 0.253 | 0.833 | 15 |
|  |  | Ketogenic | -0.317 | 1.133 | 12 |
|  |  | Total | 0.000 | 1.000 | 27 |
| Grin2a | Young | Standard | 0.841 | 0.575 | 7 |
|  |  | Ketogenic | 0.365 | 0.633 | 6 |
|  |  | Total | 0.621 | 0.627 | 13 |
|  | Aged | Standard | -0.187 | 0.747 | 8 |
|  |  | Ketogenic | -1.096 | 0.986 | 6 |
|  |  | Total | -0.577 | 0.945 | 14 |
|  | Total | Standard | 0.293 | 0.838 | 15 |
|  |  | Ketogenic | -0.366 | 1.098 | 12 |
|  |  | Total | 0.000 | 1.000 | 27 |
| Grin2b | Young | Standard | 0.864 | 0.626 | 7 |
|  |  | Ketogenic | 0.314 | 0.954 | 6 |
|  |  | Total | 0.610 | 0.810 | 13 |
|  | Aged | Standard | -0.239 | 0.749 | 8 |
|  |  | Ketogenic | -1.003 | 0.755 | 6 |
|  |  | Total | -0.567 | 0.822 | 14 |
|  | Total | Standard | 0.276 | 0.879 | 15 |
|  |  | Ketogenic | -0.344 | 1.071 | 12 |
|  |  | Total | 0.000 | 1.000 | 27 |
| Grm1 | Young | Standard | 0.861 | 0.976 | 7 |
|  |  | Ketogenic | -0.145 | 0.711 | 6 |
|  |  | Total | 0.397 | 0.980 | 13 |
|  | Aged | Standard | -0.063 | 0.877 | 8 |
|  |  | Ketogenic | -0.932 | 0.649 | 5 |
|  |  | Total | -0.397 | 0.884 | 13 |
|  | Total | Standard | 0.368 | 1.010 | 15 |
|  |  | Ketogenic | -0.502 | 0.768 | 11 |
|  |  | Total | 0.000 | 1.000 | 26 |
| Grm3 | Young | Standard | 0.679 | 0.573 | 7 |
|  |  | Ketogenic | 0.074 | 0.466 | 6 |
|  |  | Total | 0.400 | 0.594 | 13 |
|  | Aged | Standard | -0.087 | 1.156 | 8 |
|  |  | Ketogenic | -0.750 | 1.175 | 6 |
|  |  | Total | -0.371 | 1.169 | 14 |
|  | Total | Standard | 0.270 | 0.982 | 15 |
|  |  | Ketogenic | -0.338 | 0.955 | 12 |
|  |  | Total | 0.000 | 1.000 | 27 |
| Grm5 | Young | Standard | 0.878 | 0.826 | 7 |
|  |  | Ketogenic | 0.142 | 1.100 | 6 |
|  |  | Total | 0.538 | 0.995 | 13 |
|  | Aged | Standard | -0.214 | 0.715 | 8 |
|  |  | Ketogenic | -0.881 | 0.590 | 6 |
|  |  | Total | -0.500 | 0.726 | 14 |
|  | Total | Standard | 0.296 | 0.930 | 15 |
|  |  | Ketogenic | -0.369 | 0.997 | 12 |
|  |  | Total | 0.000 | 1.000 | 27 |
| Grm7 | Young | Standard | 0.816 | 0.560 | 7 |
|  |  | Ketogenic | 0.328 | 0.668 | 6 |
|  |  | Total | 0.590 | 0.638 | 13 |
|  | Aged | Standard | -0.145 | 0.692 | 8 |
|  |  | Ketogenic | -1.086 | 1.094 | 6 |
|  |  | Total | -0.548 | 0.975 | 14 |
|  | Total | Standard | 0.303 | 0.787 | 15 |
|  |  | Ketogenic | -0.379 | 1.137 | 12 |
|  |  | Total | 0.000 | 1.000 | 27 |
| Homer1 | Young | Standard | 0.967 | 0.651 | 7 |
|  |  | Ketogenic | 0.164 | 0.910 | 6 |
|  |  | Total | 0.596 | 0.855 | 13 |
|  | Aged | Standard | -0.361 | 0.736 | 8 |
|  |  | Ketogenic | -0.810 | 0.876 | 6 |
|  |  | Total | -0.554 | 0.800 | 14 |
|  | Total | Standard | 0.258 | 0.961 | 15 |
|  |  | Ketogenic | -0.323 | 0.992 | 12 |
|  |  | Total | 0.000 | 1.000 | 27 |
| Itpr1 | Young | Standard | 0.980 | 0.746 | 7 |
|  |  | Ketogenic | 0.116 | 1.071 | 6 |
|  |  | Total | 0.581 | 0.978 | 13 |
|  | Aged | Standard | -0.377 | 0.762 | 8 |
|  |  | Ketogenic | -0.757 | 0.544 | 6 |
|  |  | Total | -0.540 | 0.681 | 14 |
|  | Total | Standard | 0.256 | 1.010 | 15 |
|  |  | Ketogenic | -0.320 | 0.929 | 12 |
|  |  | Total | 0.000 | 1.000 | 27 |
| Mapk1 | Young | Standard | 0.645 | 0.410 | 7 |
|  |  | Ketogenic | 0.232 | 0.684 | 6 |
|  |  | Total | 0.454 | 0.570 | 13 |
|  | Aged | Standard | -0.256 | 1.162 | 8 |
|  |  | Ketogenic | -0.643 | 1.180 | 6 |
|  |  | Total | -0.422 | 1.141 | 14 |
|  | Total | Standard | 0.164 | 0.982 | 15 |
|  |  | Ketogenic | -0.205 | 1.027 | 12 |
|  |  | Total | 0.000 | 1.000 | 27 |
| Nsf | Young | Standard | 1.099 | 0.485 | 7 |
|  |  | Ketogenic | -0.010 | 0.757 | 6 |
|  |  | Total | 0.587 | 0.829 | 13 |
|  | Aged | Standard | -0.177 | 0.578 | 8 |
|  |  | Ketogenic | -1.035 | 0.922 | 6 |
|  |  | Total | -0.545 | 0.837 | 14 |
|  | Total | Standard | 0.418 | 0.838 | 15 |
|  |  | Ketogenic | -0.523 | 0.967 | 12 |
|  |  | Total | 0.000 | 1.000 | 27 |
| P2rx7 | Young | Standard | 0.264 | 0.934 | 7 |
|  |  | Ketogenic | -0.552 | 0.845 | 6 |
|  |  | Total | -0.113 | 0.955 | 13 |
|  | Aged | Standard | 0.240 | 1.144 | 8 |
|  |  | Ketogenic | -0.113 | 1.035 | 4 |
|  |  | Total | 0.122 | 1.075 | 12 |
|  | Total | Standard | 0.251 | 1.014 | 15 |
|  |  | Ketogenic | -0.376 | 0.897 | 10 |
|  |  | Total | 0.000 | 1.000 | 25 |
| Phgdh | Young | Standard | 0.707 | 1.153 | 7 |
|  |  | Ketogenic | -0.282 | 0.745 | 6 |
|  |  | Total | 0.250 | 1.077 | 13 |
|  | Aged | Standard | 0.136 | 0.868 | 8 |
|  |  | Ketogenic | -0.724 | 0.735 | 6 |
|  |  | Total | -0.233 | 0.899 | 14 |
|  | Total | Standard | 0.402 | 1.017 | 15 |
|  |  | Ketogenic | -0.503 | 0.743 | 12 |
|  |  | Total | 0.000 | 1.000 | 27 |
| Pla2g6 | Young | Standard | 0.963 | 0.936 | 7 |
|  |  | Ketogenic | -0.087 | 0.659 | 6 |
|  |  | Total | 0.479 | 0.957 | 13 |
|  | Aged | Standard | 0.023 | 0.397 | 8 |
|  |  | Ketogenic | -1.068 | 0.904 | 6 |
|  |  | Total | -0.444 | 0.844 | 14 |
|  | Total | Standard | 0.462 | 0.831 | 15 |
|  |  | Ketogenic | -0.577 | 0.912 | 12 |
|  |  | Total | 0.000 | 1.000 | 27 |
| Plcb1 | Young | Standard | 0.980 | 0.519 | 7 |
|  |  | Ketogenic | 0.085 | 0.521 | 6 |
|  |  | Total | 0.567 | 0.681 | 13 |
|  | Aged | Standard | -0.403 | 1.019 | 8 |
|  |  | Ketogenic | -0.690 | 0.984 | 6 |
|  |  | Total | -0.526 | 0.976 | 14 |
|  | Total | Standard | 0.242 | 1.070 | 15 |
|  |  | Ketogenic | -0.303 | 0.853 | 12 |
|  |  | Total | 0.000 | 1.000 | 27 |
| Prodh | Young | Standard | 0.551 | 1.000 | 7 |
|  |  | Ketogenic | 0.237 | 1.000 | 6 |
|  |  | Total | 0.406 | 0.971 | 13 |
|  | Aged | Standard | 0.074 | 0.831 | 8 |
|  |  | Ketogenic | -0.978 | 0.618 | 6 |
|  |  | Total | -0.377 | 0.900 | 14 |
|  | Total | Standard | 0.297 | 0.914 | 15 |
|  |  | Ketogenic | -0.371 | 1.015 | 12 |
|  |  | Total | 0.000 | 1.000 | 27 |
| Shank2 | Young | Standard | 0.872 | 0.995 | 7 |
|  |  | Ketogenic | 0.254 | 0.859 | 6 |
|  |  | Total | 0.587 | 0.951 | 13 |
|  | Aged | Standard | -0.292 | 0.511 | 8 |
|  |  | Ketogenic | -0.883 | 0.833 | 6 |
|  |  | Total | -0.545 | 0.707 | 14 |
|  | Total | Standard | 0.251 | 0.957 | 15 |
|  |  | Ketogenic | -0.314 | 1.002 | 12 |
|  |  | Total | 0.000 | 1.000 | 27 |
| Slc17a7 | Young | Standard | 0.804 | 0.970 | 7 |
|  |  | Ketogenic | 0.161 | 0.935 | 6 |
|  |  | Total | 0.507 | 0.973 | 13 |
|  | Aged | Standard | -0.216 | 0.931 | 8 |
|  |  | Ketogenic | -0.812 | 0.428 | 6 |
|  |  | Total | -0.471 | 0.794 | 14 |
|  | Total | Standard | 0.260 | 1.055 | 15 |
|  |  | Ketogenic | -0.325 | 0.860 | 12 |
|  |  | Total | 0.000 | 1.000 | 27 |
| Slc1a1 | Young | Standard | 0.445 | 0.897 | 7 |
|  |  | Ketogenic | 0.627 | 0.990 | 6 |
|  |  | Total | 0.529 | 0.906 | 13 |
|  | Aged | Standard | -0.158 | 0.493 | 8 |
|  |  | Ketogenic | -0.936 | 1.036 | 6 |
|  |  | Total | -0.491 | 0.839 | 14 |
|  | Total | Standard | 0.124 | 0.751 | 15 |
|  |  | Ketogenic | -0.155 | 1.265 | 12 |
|  |  | Total | 0.000 | 1.000 | 27 |
| Slc1a2 | Young | Standard | 0.418 | 0.600 | 7 |
|  |  | Ketogenic | 0.244 | 0.507 | 6 |
|  |  | Total | 0.337 | 0.543 | 13 |
|  | Aged | Standard | 0.126 | 1.112 | 8 |
|  |  | Ketogenic | -0.899 | 1.211 | 6 |
|  |  | Total | -0.313 | 1.228 | 14 |
|  | Total | Standard | 0.262 | 0.892 | 15 |
|  |  | Ketogenic | -0.328 | 1.068 | 12 |
|  |  | Total | 0.000 | 1.000 | 27 |
| Slc1a3 | Young | Standard | 0.611 | 0.497 | 7 |
|  |  | Ketogenic | -0.328 | 0.576 | 6 |
|  |  | Total | 0.177 | 0.707 | 13 |
|  | Aged | Standard | 0.294 | 1.089 | 8 |
|  |  | Ketogenic | -0.776 | 1.182 | 6 |
|  |  | Total | -0.165 | 1.216 | 14 |
|  | Total | Standard | 0.442 | 0.852 | 15 |
|  |  | Ketogenic | -0.552 | 0.917 | 12 |
|  |  | Total | 0.000 | 1.000 | 27 |
| Slc38a1 | Young | Standard | 0.956 | 0.719 | 7 |
|  |  | Ketogenic | -0.075 | 0.762 | 6 |
|  |  | Total | 0.480 | 0.887 | 13 |
|  | Aged | Standard | -0.063 | 0.397 | 8 |
|  |  | Ketogenic | -0.957 | 1.177 | 6 |
|  |  | Total | -0.446 | 0.910 | 14 |
|  | Total | Standard | 0.413 | 0.760 | 15 |
|  |  | Ketogenic | -0.516 | 1.052 | 12 |
|  |  | Total | 0.000 | 1.000 | 27 |
| Slc6a1 | Young | Standard | 1.277 | 0.712 | 7 |
|  |  | Ketogenic | -0.188 | 0.667 | 6 |
|  |  | Total | 0.601 | 1.009 | 13 |
|  | Aged | Standard | -0.352 | 0.463 | 8 |
|  |  | Ketogenic | -0.833 | 0.700 | 6 |
|  |  | Total | -0.558 | 0.604 | 14 |
|  | Total | Standard | 0.408 | 1.016 | 15 |
|  |  | Ketogenic | -0.511 | 0.733 | 12 |
|  |  | Total | 0.000 | 1.000 | 27 |
| Slc6a11 | Young | Standard | 0.954 | 0.615 | 7 |
|  |  | Ketogenic | 0.249 | 0.798 | 6 |
|  |  | Total | 0.628 | 0.767 | 13 |
|  | Aged | Standard | -0.186 | 0.469 | 8 |
|  |  | Ketogenic | -1.113 | 0.955 | 6 |
|  |  | Total | -0.583 | 0.834 | 14 |
|  | Total | Standard | 0.346 | 0.786 | 15 |
|  |  | Ketogenic | -0.432 | 1.100 | 12 |
|  |  | Total | 0.000 | 1.000 | 27 |
| Snca | Young | Standard | 0.886 | 0.490 | 7 |
|  |  | Ketogenic | 0.581 | 0.895 | 6 |
|  |  | Total | 0.746 | 0.692 | 13 |
|  | Aged | Standard | -0.516 | 0.545 | 8 |
|  |  | Ketogenic | -0.927 | 0.855 | 6 |
|  |  | Total | -0.692 | 0.697 | 14 |
|  | Total | Standard | 0.138 | 0.881 | 15 |
|  |  | Ketogenic | -0.173 | 1.148 | 12 |
|  |  | Total | 0.000 | 1.000 | 27 |
| Srr | Young | Standard | 0.921 | 0.533 | 7 |
|  |  | Ketogenic | 0.300 | 0.800 | 6 |
|  |  | Total | 0.634 | 0.716 | 13 |
|  | Aged | Standard | -0.257 | 0.722 | 8 |
|  |  | Ketogenic | -1.032 | 0.900 | 6 |
|  |  | Total | -0.589 | 0.866 | 14 |
|  | Total | Standard | 0.293 | 0.867 | 15 |
|  |  | Ketogenic | -0.366 | 1.069 | 12 |
|  |  | Total | 0.000 | 1.000 | 27 |

Table S5: Descriptive statistics for gene expression within CA3.

| Effect | Gene | Type III Sum of Squares | df | Mean Square | F | p-values | Partial Eta Squared | Noncent. Parameter | Observed Power^bz^ | Rank | FDR q-values 0.5% |
| --- | --- | --- | --- | --- | --- | --- | --- | --- | --- | --- | --- |
| Age | Abat | 4.267 | 1 | 4.267 | 6.680 | 0.017 | 0.225 | 6.680 | 0.697 | 45 | 0.004 |
|  | Adora1 | 5.786 | 1 | 5.786 | 8.464 | 0.008 | 0.269 | 8.464 | 0.796 | 38 | 0.003 |
|  | Aldh5a1 | 5.495 | 1 | 5.495 | 9.181 | 0.006 | 0.285 | 9.181 | 0.827 | 35 | 0.003 |
|  | App | 9.969 | 1 | 9.969 | 19.310 | 0.000 | 0.456 | 19.310 | 0.988 | 6 | 0.001 |
|  | Bdnf | 9.554 | 1 | 9.554 | 10.856 | 0.003 | 0.330 | 10.856 | 0.883 | 41 | 0.004 |
|  | Cacna1a | 5.941 | 1 | 5.941 | 8.073 | 0.009 | 0.260 | 8.073 | 0.777 | 39 | 0.003 |
|  | Cdk5r1 | 7.651 | 1 | 7.651 | 12.951 | 0.002 | 0.360 | 12.951 | 0.931 | 22 | 0.002 |
|  | Dlg4 | 4.537 | 1 | 4.537 | 7.198 | 0.013 | 0.238 | 7.198 | 0.729 | 42 | 0.004 |
|  | Gabbr1 | 2.894 | 1 | 2.894 | 3.590 | 0.071 | 0.135 | 3.590 | 0.443 | 54 | 0.005 |
|  | Gabbr2 | 8.338 | 1 | 8.338 | 13.929 | 0.001 | 0.377 | 13.929 | 0.947 | 18 | 0.002 |
|  | Gabra1 | 5.234 | 1 | 5.234 | 6.096 | 0.021 | 0.210 | 6.096 | 0.657 | 49 | 0.004 |
|  | Gabra2 | 8.274 | 1 | 8.274 | 11.180 | 0.003 | 0.327 | 11.180 | 0.893 | 27 | 0.002 |
|  | Gabra4 | 5.073 | 1 | 5.073 | 7.147 | 0.014 | 0.237 | 7.147 | 0.726 | 43 | 0.004 |
|  | Gabra5 | 9.346 | 1 | 9.346 | 16.766 | 0.000 | 0.422 | 16.766 | 0.975 | 12 | 0.001 |
|  | Gabrb1 | 6.866 | 1 | 6.866 | 9.580 | 0.005 | 0.294 | 9.580 | 0.842 | 34 | 0.003 |
|  | Gabrb3 | 8.337 | 1 | 8.337 | 12.269 | 0.002 | 0.348 | 12.269 | 0.918 | 23 | 0.002 |
|  | Gabrg1 | 4.196 | 1 | 4.196 | 5.249 | 0.031 | 0.186 | 5.249 | 0.593 | 51 | 0.004 |
|  | Gabrg2 | 7.901 | 1 | 7.901 | 11.045 | 0.003 | 0.324 | 11.045 | 0.889 | 29 | 0.003 |
|  | Gabrg3 | 29.087 | 1 | 29.087 | 14.343 | 0.001 | 0.384 | 14.343 | 0.952 | 24 | 0.002 |
|  | Gad1 | 7.721 | 1 | 7.721 | 10.683 | 0.004 | 0.327 | 10.683 | 0.878 | 31 | 0.003 |
|  | Gls | 9.630 | 1 | 9.630 | 17.415 | 0.000 | 0.431 | 17.415 | 0.979 | 11 | 0.001 |
|  | Glul | 1.470 | 1 | 1.470 | 2.127 | 0.158 | 0.085 | 2.127 | 0.287 | 55 | 0.005 |
|  | Gnai1 | 9.657 | 1 | 9.657 | 14.319 | 0.001 | 0.384 | 14.319 | 0.952 | 16 | 0.001 |
|  | Gnaq | 9.590 | 1 | 9.590 | 17.480 | 0.000 | 0.432 | 17.480 | 0.979 | 10 | 0.001 |
|  | Gphn | 8.957 | 1 | 8.957 | 14.569 | 0.001 | 0.388 | 14.569 | 0.955 | 15 | 0.001 |
|  | Gria1 | 5.950 | 1 | 5.950 | 8.907 | 0.007 | 0.279 | 8.907 | 0.815 | 37 | 0.003 |
|  | Gria2 | 4.718 | 1 | 4.718 | 5.632 | 0.026 | 0.197 | 5.632 | 0.623 | 50 | 0.004 |
|  | Gria3 | 5.723 | 1 | 5.723 | 7.610 | 0.011 | 0.249 | 7.610 | 0.752 | 40 | 0.003 |
|  | Gria4 | 5.329 | 1 | 5.329 | 6.949 | 0.015 | 0.232 | 6.949 | 0.714 | 44 | 0.004 |
|  | Grik2 | 9.215 | 1 | 9.215 | 13.544 | 0.001 | 0.371 | 13.544 | 0.941 | 20 | 0.002 |
|  | Grik4 | 9.853 | 1 | 9.853 | 19.463 | 0.000 | 0.458 | 19.463 | 0.988 | 5 | 0.000 |
|  | Grik5 | 6.742 | 1 | 6.742 | 9.954 | 0.004 | 0.302 | 9.954 | 0.856 | 32 | 0.003 |
|  | Grin1 | 11.280 | 1 | 11.280 | 21.037 | 0.000 | 0.478 | 21.037 | 0.992 | 3 | 0.000 |
|  | Grin2a | 10.304 | 1 | 10.304 | 18.569 | 0.000 | 0.447 | 18.569 | 0.985 | 9 | 0.001 |
|  | Grin2b | 9.746 | 1 | 9.746 | 16.378 | 0.001 | 0.416 | 16.378 | 0.972 | 13 | 0.001 |
|  | Grm1 | 4.615 | 1 | 4.615 | 6.633 | 0.017 | 0.232 | 6.633 | 0.692 | 46 | 0.004 |
|  | Grm3 | 4.206 | 1 | 4.206 | 5.012 | 0.035 | 0.179 | 5.012 | 0.573 | 52 | 0.004 |
|  | Grm5 | 7.444 | 1 | 7.444 | 11.076 | 0.003 | 0.325 | 11.076 | 0.890 | 28 | 0.002 |
|  | Grm7 | 9.378 | 1 | 9.378 | 16.032 | 0.001 | 0.411 | 16.032 | 0.969 | 14 | 0.001 |
|  | Homer1 | 8.815 | 1 | 8.815 | 14.162 | 0.001 | 0.381 | 14.162 | 0.950 | 17 | 0.001 |
|  | Itpr1 | 8.277 | 1 | 8.277 | 13.021 | 0.001 | 0.361 | 13.021 | 0.932 | 21 | 0.002 |
|  | Mapk1 | 5.249 | 1 | 5.249 | 6.109 | 0.021 | 0.210 | 6.109 | 0.658 | 48 | 0.004 |
|  | Nsf | 8.812 | 1 | 8.812 | 18.648 | 0.000 | 0.448 | 18.648 | 0.985 | 8 | 0.001 |
|  | P2rx7 | 0.251 | 1 | 0.251 | 0.249 | 0.623 | 0.012 | 0.249 | 0.076 | 58 | 0.005 |
|  | Phgdh | 1.707 | 1 | 1.707 | 2.096 | 0.161 | 0.084 | 2.096 | 0.284 | 56 | 0.005 |
|  | Pla2g6 | 6.138 | 1 | 6.138 | 11.188 | 0.003 | 0.327 | 11.188 | 0.893 | 26 | 0.002 |
|  | Plcb1 | 7.743 | 1 | 7.743 | 11.809 | 0.002 | 0.339 | 11.809 | 0.908 | 25 | 0.002 |
|  | Prodh | 4.769 | 1 | 4.769 | 6.180 | 0.021 | 0.212 | 6.180 | 0.663 | 47 | 0.004 |
|  | Shank2 | 8.809 | 1 | 8.809 | 13.572 | 0.001 | 0.371 | 13.572 | 0.941 | 19 | 0.002 |
|  | Slc17a7 | 6.601 | 1 | 6.601 | 8.931 | 0.007 | 0.280 | 8.931 | 0.816 | 36 | 0.003 |
|  | Slc1a1 | 7.795 | 1 | 7.795 | 10.667 | 0.003 | 0.317 | 10.667 | 0.878 | 30 | 0.003 |
|  | Slc1a2 | 3.424 | 1 | 3.424 | 4.050 | 0.056 | 0.150 | 4.050 | 0.487 | 53 | 0.005 |
|  | Slc1a3 | 0.972 | 1 | 0.972 | 1.212 | 0.282 | 0.050 | 1.212 | 0.184 | 57 | 0.005 |
|  | Slc38a1 | 6.012 | 1 | 6.012 | 9.854 | 0.005 | 0.300 | 9.854 | 0.852 | 33 | 0.003 |
|  | Slc6a1 | 8.599 | 1 | 8.599 | 21.467 | 0.000 | 0.483 | 21.467 | 0.993 | 2 | 0.000 |
|  | Slc6a11 | 10.408 | 1 | 10.408 | 20.723 | 0.000 | 0.474 | 20.723 | 0.992 | 4 | 0.000 |
|  | Snca | 14.096 | 1 | 14.096 | 28.988 | 0.000 | 0.558 | 28.988 | 0.999 | 1 | 0.000 |
|  | Srr | 10.481 | 1 | 10.481 | 19.133 | 0.000 | 0.454 | 19.133 | 0.987 | 7 | 0.001 |
| Diet | Abat | 7.275 | 1 | 7.275 | 11.388 | 0.003 | 0.331 | 11.388 | 0.898 | 4 | 0.000 |
|  | Adora1 | 3.487 | 1 | 3.487 | 5.101 | 0.034 | 0.182 | 5.101 | 0.581 | 27 | 0.002 |
|  | Aldh5a1 | 6.492 | 1 | 6.492 | 10.848 | 0.003 | 0.320 | 10.848 | 0.884 | 5 | 0.000 |
|  | App | 4.351 | 1 | 4.351 | 8.428 | 0.008 | 0.268 | 8.428 | 0.794 | 10 | 0.001 |
|  | Bdnf | 2.394 | 1 | 2.394 | 2.720 | 0.113 | 0.110 | 2.720 | 0.351 | 54 | 0.005 |
|  | Cacna1a | 2.592 | 1 | 2.592 | 3.522 | 0.073 | 0.133 | 3.522 | 0.436 | 39 | 0.003 |
|  | Cdk5r1 | 4.255 | 1 | 4.255 | 7.202 | 0.013 | 0.238 | 7.202 | 0.729 | 16 | 0.001 |
|  | Dlg4 | 6.403 | 1 | 6.403 | 10.158 | 0.004 | 0.306 | 10.158 | 0.862 | 6 | 0.001 |
|  | Gabbr1 | 4.166 | 1 | 4.166 | 5.169 | 0.033 | 0.183 | 5.169 | 0.586 | 26 | 0.002 |
|  | Gabbr2 | 3.872 | 1 | 3.872 | 6.468 | 0.018 | 0.219 | 6.468 | 0.683 | 19 | 0.002 |
|  | Gabra1 | 0.030 | 1 | 0.030 | 0.035 | 0.853 | 0.002 | 0.035 | 0.054 | 57 | 0.005 |
|  | Gabra2 | 0.784 | 1 | 0.784 | 1.059 | 0.314 | 0.044 | 1.059 | 0.167 | 53 | 0.005 |
|  | Gabra4 | 4.507 | 1 | 4.507 | 6.350 | 0.019 | 0.216 | 6.350 | 0.675 | 20 | 0.002 |
|  | Gabra5 | 4.056 | 1 | 4.056 | 7.276 | 0.013 | 0.240 | 7.276 | 0.733 | 15 | 0.001 |
|  | Gabrb1 | 0.008 | 1 | 0.008 | 0.011 | 0.918 | 0.000 | 0.011 | 0.051 | 58 | 0.005 |
|  | Gabrb3 | 0.856 | 1 | 0.856 | 1.259 | 0.273 | 0.052 | 1.259 | 0.189 | 51 | 0.004 |
|  | Gabrg1 | 3.565 | 1 | 3.565 | 4.460 | 0.046 | 0.162 | 4.460 | 0.525 | 31 | 0.003 |
|  | Gabrg2 | 1.865 | 1 | 1.865 | 2.607 | 0.120 | 0.102 | 2.607 | 0.340 | 44 | 0.004 |
|  | Gabrg3 | 0.031 | 1 | 0.031 | 0.016 | 0.902 | 0.001 | 0.016 | 0.052 | 45 | 0.004 |
|  | Gad1 | 1.307 | 1 | 1.307 | 1.809 | 0.192 | 0.076 | 1.809 | 0.251 | 48 | 0.004 |
|  | Gls | 3.766 | 1 | 3.766 | 6.810 | 0.016 | 0.228 | 6.810 | 0.705 | 18 | 0.002 |
|  | Glul | 0.151 | 1 | 0.151 | 0.218 | 0.645 | 0.009 | 0.218 | 0.073 | 56 | 0.005 |
|  | Gnai1 | 1.004 | 1 | 1.004 | 1.489 | 0.235 | 0.061 | 1.489 | 0.216 | 50 | 0.004 |
|  | Gnaq | 4.125 | 1 | 4.125 | 7.518 | 0.012 | 0.246 | 7.518 | 0.747 | 13 | 0.001 |
|  | Gphn | 3.208 | 1 | 3.208 | 5.217 | 0.032 | 0.185 | 5.217 | 0.590 | 25 | 0.002 |
|  | Gria1 | 4.878 | 1 | 4.878 | 7.302 | 0.013 | 0.241 | 7.302 | 0.735 | 14 | 0.001 |
|  | Gria2 | 2.199 | 1 | 2.199 | 2.624 | 0.119 | 0.102 | 2.624 | 0.342 | 43 | 0.004 |
|  | Gria3 | 3.215 | 1 | 3.215 | 4.275 | 0.050 | 0.157 | 4.275 | 0.508 | 32 | 0.003 |
|  | Gria4 | 3.226 | 1 | 3.226 | 4.206 | 0.052 | 0.155 | 4.206 | 0.502 | 33 | 0.003 |
|  | Grik2 | 1.276 | 1 | 1.276 | 1.875 | 0.184 | 0.075 | 1.875 | 0.259 | 47 | 0.004 |
|  | Grik4 | 4.838 | 1 | 4.838 | 9.557 | 0.005 | 0.294 | 9.557 | 0.841 | 8 | 0.001 |
|  | Grik5 | 3.867 | 1 | 3.867 | 5.709 | 0.025 | 0.199 | 5.709 | 0.629 | 24 | 0.002 |
|  | Grin1 | 2.420 | 1 | 2.420 | 4.513 | 0.045 | 0.164 | 4.513 | 0.530 | 30 | 0.003 |
|  | Grin2a | 3.192 | 1 | 3.192 | 5.753 | 0.025 | 0.200 | 5.753 | 0.632 | 23 | 0.002 |
|  | Grin2b | 2.871 | 1 | 2.871 | 4.824 | 0.038 | 0.173 | 4.824 | 0.557 | 29 | 0.003 |
|  | Grm1 | 5.540 | 1 | 5.540 | 7.961 | 0.010 | 0.266 | 7.961 | 0.769 | 12 | 0.001 |
|  | Grm3 | 2.676 | 1 | 2.676 | 3.188 | 0.087 | 0.122 | 3.188 | 0.402 | 41 | 0.004 |
|  | Grm5 | 3.274 | 1 | 3.274 | 4.871 | 0.038 | 0.175 | 4.871 | 0.561 | 28 | 0.002 |
|  | Grm7 | 3.396 | 1 | 3.396 | 5.805 | 0.024 | 0.202 | 5.805 | 0.636 | 22 | 0.002 |
|  | Homer1 | 2.606 | 1 | 2.606 | 4.187 | 0.052 | 0.154 | 4.187 | 0.500 | 34 | 0.003 |
|  | Itpr1 | 2.569 | 1 | 2.569 | 4.042 | 0.056 | 0.149 | 4.042 | 0.487 | 35 | 0.003 |
|  | Mapk1 | 1.063 | 1 | 1.063 | 1.237 | 0.278 | 0.051 | 1.237 | 0.187 | 52 | 0.004 |
|  | Nsf | 6.430 | 1 | 6.430 | 13.607 | 0.001 | 0.372 | 13.607 | 0.942 | 3 | 0.000 |
|  | P2rx7 | 1.996 | 1 | 1.996 | 1.979 | 0.174 | 0.086 | 1.979 | 0.269 | 46 | 0.004 |
|  | Phgdh | 5.688 | 1 | 5.688 | 6.985 | 0.015 | 0.233 | 6.985 | 0.716 | 17 | 0.001 |
|  | Pla2g6 | 7.621 | 1 | 7.621 | 13.891 | 0.001 | 0.377 | 13.891 | 0.946 | 2 | 0.000 |
|  | Plcb1 | 2.325 | 1 | 2.325 | 3.546 | 0.072 | 0.134 | 3.546 | 0.438 | 38 | 0.003 |
|  | Prodh | 3.105 | 1 | 3.105 | 4.024 | 0.057 | 0.149 | 4.024 | 0.485 | 36 | 0.003 |
|  | Shank2 | 2.432 | 1 | 2.432 | 3.747 | 0.065 | 0.140 | 3.747 | 0.458 | 37 | 0.003 |
|  | Slc17a7 | 2.550 | 1 | 2.550 | 3.450 | 0.076 | 0.130 | 3.450 | 0.429 | 40 | 0.003 |
|  | Slc1a1 | 0.592 | 1 | 0.592 | 0.810 | 0.377 | 0.034 | 0.810 | 0.139 | 55 | 0.005 |
|  | Slc1a2 | 2.393 | 1 | 2.393 | 2.830 | 0.106 | 0.110 | 2.830 | 0.364 | 42 | 0.004 |
|  | Slc1a3 | 6.717 | 1 | 6.717 | 8.381 | 0.008 | 0.267 | 8.381 | 0.792 | 11 | 0.001 |
|  | Slc38a1 | 6.173 | 1 | 6.173 | 10.117 | 0.004 | 0.305 | 10.117 | 0.861 | 7 | 0.001 |
|  | Slc6a1 | 6.303 | 1 | 6.303 | 15.734 | 0.001 | 0.406 | 15.734 | 0.967 | 1 | 0.000 |
|  | Slc6a11 | 4.432 | 1 | 4.432 | 8.825 | 0.007 | 0.277 | 8.825 | 0.812 | 9 | 0.001 |
|  | Snca | 0.852 | 1 | 0.852 | 1.752 | 0.199 | 0.071 | 1.752 | 0.245 | 49 | 0.004 |
|  | Srr | 3.244 | 1 | 3.244 | 5.922 | 0.023 | 0.205 | 5.922 | 0.645 | 21 | 0.002 |
| Age * Diet | Abat | 0.185 | 1 | 0.185 | 0.290 | 0.596 | 0.012 | 0.290 | 0.081 | 33 | 0.003 |
|  | Adora1 | 1.740 | 1 | 1.740 | 2.545 | 0.124 | 0.100 | 2.545 | 0.333 | 6 | 0.001 |
|  | Aldh5a1 | 0.882 | 1 | 0.882 | 1.474 | 0.237 | 0.060 | 1.474 | 0.214 | 12 | 0.001 |
|  | App | 0.498 | 1 | 0.498 | 0.964 | 0.336 | 0.040 | 0.964 | 0.156 | 18 | 0.002 |
|  | Bdnf | 4.799 | 1 | 4.799 | 5.453 | 0.029 | 0.199 | 5.453 | 0.607 | 5 | 0.000 |
|  | Cacna1a | 1.176 | 1 | 1.176 | 1.598 | 0.219 | 0.065 | 1.598 | 0.228 | 11 | 0.001 |
|  | Cdk5r1 | 1.307 | 1 | 1.307 | 2.213 | 0.150 | 0.088 | 2.213 | 0.297 | 7 | 0.001 |
|  | Dlg4 | 1.164 | 1 | 1.164 | 1.846 | 0.187 | 0.074 | 1.846 | 0.256 | 9 | 0.001 |
|  | Gabbr1 | 0.792 | 1 | 0.792 | 0.983 | 0.332 | 0.041 | 0.983 | 0.158 | 17 | 0.001 |
|  | Gabbr2 | 0.706 | 1 | 0.706 | 1.180 | 0.289 | 0.049 | 1.180 | 0.180 | 15 | 0.001 |
|  | Gabra1 | 1.545 | 1 | 1.545 | 1.800 | 0.193 | 0.073 | 1.800 | 0.251 | 10 | 0.001 |
|  | Gabra2 | 0.288 | 1 | 0.288 | 0.389 | 0.539 | 0.017 | 0.389 | 0.092 | 29 | 0.003 |
|  | Gabra4 | 0.610 | 1 | 0.610 | 0.859 | 0.364 | 0.036 | 0.859 | 0.144 | 20 | 0.002 |
|  | Gabra5 | 0.398 | 1 | 0.398 | 0.714 | 0.407 | 0.030 | 0.714 | 0.128 | 21 | 0.002 |
|  | Gabrb1 | 3.620 | 1 | 3.620 | 5.051 | 0.034 | 0.180 | 5.051 | 0.577 | 2 | 0.000 |
|  | Gabrb3 | 2.052 | 1 | 2.052 | 3.020 | 0.096 | 0.116 | 3.020 | 0.384 | 4 | 0.000 |
|  | Gabrg1 | 0.013 | 1 | 0.013 | 0.016 | 0.901 | 0.001 | 0.016 | 0.052 | 49 | 0.004 |
|  | Gabrg2 | 0.064 | 1 | 0.064 | 0.089 | 0.768 | 0.004 | 0.089 | 0.059 | 40 | 0.003 |
|  | Gabrg3 | 2.935 | 1 | 2.935 | 1.447 | 0.241 | 0.059 | 1.447 | 0.211 | 25 | 0.002 |
|  | Gad1 | 0.003 | 1 | 0.003 | 0.004 | 0.951 | 0.000 | 0.004 | 0.050 | 54 | 0.005 |
|  | Gls | 0.041 | 1 | 0.041 | 0.074 | 0.787 | 0.003 | 0.074 | 0.058 | 41 | 0.004 |
|  | Glul | 9.116 | 1 | 9.116 | 13.192 | 0.001 | 0.364 | 13.192 | 0.935 | 1 | 0.000 |
|  | Gnai1 | 0.128 | 1 | 0.128 | 0.190 | 0.667 | 0.008 | 0.190 | 0.070 | 35 | 0.003 |
|  | Gnaq | 0.194 | 1 | 0.194 | 0.354 | 0.558 | 0.015 | 0.354 | 0.088 | 30 | 0.003 |
|  | Gphn | 0.110 | 1 | 0.110 | 0.178 | 0.677 | 0.008 | 0.178 | 0.069 | 36 | 0.003 |
|  | Gria1 | 0.290 | 1 | 0.290 | 0.434 | 0.516 | 0.019 | 0.434 | 0.097 | 27 | 0.002 |
|  | Gria2 | 0.032 | 1 | 0.032 | 0.038 | 0.848 | 0.002 | 0.038 | 0.054 | 46 | 0.004 |
|  | Gria3 | 0.095 | 1 | 0.095 | 0.127 | 0.725 | 0.005 | 0.127 | 0.063 | 39 | 0.003 |
|  | Gria4 | 0.000 | 1 | 0.000 | 0.000 | 0.986 | 0.000 | 0.000 | 0.050 | 58 | 0.005 |
|  | Grik2 | 0.282 | 1 | 0.282 | 0.414 | 0.526 | 0.018 | 0.414 | 0.095 | 28 | 0.002 |
|  | Grik4 | 0.001 | 1 | 0.001 | 0.001 | 0.975 | 0.000 | 0.001 | 0.050 | 57 | 0.005 |
|  | Grik5 | 0.298 | 1 | 0.298 | 0.439 | 0.514 | 0.019 | 0.439 | 0.097 | 26 | 0.002 |
|  | Grin1 | 0.689 | 1 | 0.689 | 1.285 | 0.269 | 0.053 | 1.285 | 0.192 | 14 | 0.001 |
|  | Grin2a | 0.312 | 1 | 0.312 | 0.563 | 0.461 | 0.024 | 0.563 | 0.111 | 24 | 0.002 |
|  | Grin2b | 0.076 | 1 | 0.076 | 0.128 | 0.724 | 0.006 | 0.128 | 0.064 | 38 | 0.003 |
|  | Grm1 | 0.029 | 1 | 0.029 | 0.042 | 0.839 | 0.002 | 0.042 | 0.054 | 44 | 0.004 |
|  | Grm3 | 0.006 | 1 | 0.006 | 0.007 | 0.935 | 0.000 | 0.007 | 0.051 | 51 | 0.004 |
|  | Grm5 | 0.008 | 1 | 0.008 | 0.012 | 0.915 | 0.001 | 0.012 | 0.051 | 50 | 0.004 |
|  | Grm7 | 0.341 | 1 | 0.341 | 0.583 | 0.453 | 0.025 | 0.583 | 0.113 | 23 | 0.002 |
|  | Homer1 | 0.208 | 1 | 0.208 | 0.333 | 0.569 | 0.014 | 0.333 | 0.086 | 31 | 0.003 |
|  | Itpr1 | 0.390 | 1 | 0.390 | 0.614 | 0.441 | 0.026 | 0.614 | 0.117 | 22 | 0.002 |
|  | Mapk1 | 0.001 | 1 | 0.001 | 0.001 | 0.971 | 0.000 | 0.001 | 0.050 | 56 | 0.005 |
|  | Nsf | 0.104 | 1 | 0.104 | 0.220 | 0.643 | 0.009 | 0.220 | 0.074 | 34 | 0.003 |
|  | P2rx7 | 0.313 | 1 | 0.313 | 0.310 | 0.584 | 0.015 | 0.310 | 0.083 | 32 | 0.003 |
|  | Phgdh | 0.028 | 1 | 0.028 | 0.034 | 0.855 | 0.001 | 0.034 | 0.054 | 48 | 0.004 |
|  | Pla2g6 | 0.003 | 1 | 0.003 | 0.005 | 0.943 | 0.000 | 0.005 | 0.051 | 52 | 0.004 |
|  | Plcb1 | 0.614 | 1 | 0.614 | 0.936 | 0.343 | 0.039 | 0.936 | 0.153 | 19 | 0.002 |
|  | Prodh | 0.905 | 1 | 0.905 | 1.173 | 0.290 | 0.049 | 1.173 | 0.180 | 16 | 0.001 |
|  | Shank2 | 0.001 | 1 | 0.001 | 0.002 | 0.966 | 0.000 | 0.002 | 0.050 | 55 | 0.005 |
|  | Slc17a7 | 0.004 | 1 | 0.004 | 0.005 | 0.945 | 0.000 | 0.005 | 0.051 | 53 | 0.005 |
|  | Slc1a1 | 1.532 | 1 | 1.532 | 2.096 | 0.161 | 0.084 | 2.096 | 0.284 | 8 | 0.001 |
|  | Slc1a2 | 1.205 | 1 | 1.205 | 1.425 | 0.245 | 0.058 | 1.425 | 0.208 | 13 | 0.001 |
|  | Slc1a3 | 0.029 | 1 | 0.029 | 0.036 | 0.852 | 0.002 | 0.036 | 0.054 | 47 | 0.004 |
|  | Slc38a1 | 0.031 | 1 | 0.031 | 0.051 | 0.823 | 0.002 | 0.051 | 0.055 | 43 | 0.004 |
|  | Slc6a1 | 1.614 | 1 | 1.614 | 4.028 | 0.057 | 0.149 | 4.028 | 0.485 | 3 | 0.000 |
|  | Slc6a11 | 0.082 | 1 | 0.082 | 0.163 | 0.690 | 0.007 | 0.163 | 0.067 | 37 | 0.003 |
|  | Snca | 0.019 | 1 | 0.019 | 0.038 | 0.846 | 0.002 | 0.038 | 0.054 | 45 | 0.004 |
|  | Srr | 0.040 | 1 | 0.040 | 0.072 | 0.791 | 0.003 | 0.072 | 0.058 | 42 | 0.004 |

Table S6: Statisical summary for CA3 gene expression.

| Gene | Age | Diet | Mean (Z-Scores) | Std. Deviation | N |
| --- | --- | --- | --- | --- | --- |
| Abat | Young | Standard | 0.501 | 0.852 | 6 |
|  |  | Ketogenic | 0.107 | 1.284 | 8 |
|  |  | Total | 0.276 | 1.099 | 14 |
|  | Aged | Standard | -0.101 | 0.761 | 6 |
|  |  | Ketogenic | -0.814 | 0.456 | 4 |
|  |  | Total | -0.386 | 0.726 | 10 |
|  | Total | Standard | 0.200 | 0.832 | 12 |
|  |  | Ketogenic | -0.200 | 1.145 | 12 |
|  |  | Total | 0.000 | 1.000 | 24 |
| Adora1 | Young | Standard | 0.196 | 0.749 | 6 |
|  |  | Ketogenic | 0.247 | 1.319 | 8 |
|  |  | Total | 0.225 | 1.074 | 14 |
|  | Aged | Standard | -0.084 | 0.932 | 6 |
|  |  | Ketogenic | -0.662 | 0.622 | 4 |
|  |  | Total | -0.315 | 0.837 | 10 |
|  | Total | Standard | 0.056 | 0.819 | 12 |
|  |  | Ketogenic | -0.056 | 1.189 | 12 |
|  |  | Total | 0.000 | 1.000 | 24 |
| Aldh5a1 | Young | Standard | 0.352 | 0.934 | 6 |
|  |  | Ketogenic | -0.037 | 1.110 | 8 |
|  |  | Total | 0.130 | 1.019 | 14 |
|  | Aged | Standard | 0.148 | 1.087 | 6 |
|  |  | Ketogenic | -0.677 | 0.681 | 4 |
|  |  | Total | -0.182 | 0.996 | 10 |
|  | Total | Standard | 0.250 | 0.972 | 12 |
|  |  | Ketogenic | -0.250 | 1.005 | 12 |
|  |  | Total | 0.000 | 1.000 | 24 |
| App | Young | Standard | 0.510 | 0.903 | 6 |
|  |  | Ketogenic | 0.013 | 1.105 | 8 |
|  |  | Total | 0.226 | 1.018 | 14 |
|  | Aged | Standard | -0.165 | 1.075 | 6 |
|  |  | Ketogenic | -0.543 | 0.750 | 4 |
|  |  | Total | -0.316 | 0.931 | 10 |
|  | Total | Standard | 0.173 | 1.010 | 12 |
|  |  | Ketogenic | -0.173 | 1.003 | 12 |
|  |  | Total | 0.000 | 1.000 | 24 |
| Cdk5r1 | Young | Standard | 0.275 | 0.823 | 6 |
|  |  | Ketogenic | 0.117 | 1.005 | 8 |
|  |  | Total | 0.185 | 0.900 | 14 |
|  | Aged | Standard | 0.014 | 1.272 | 6 |
|  |  | Ketogenic | -0.667 | 0.840 | 4 |
|  |  | Total | -0.258 | 1.121 | 10 |
|  | Total | Standard | 0.144 | 1.030 | 12 |
|  |  | Ketogenic | -0.144 | 0.992 | 12 |
|  |  | Total | 0.000 | 1.000 | 24 |
| Dlg4 | Young | Standard | 0.285 | 0.658 | 5 |
|  |  | Ketogenic | 0.397 | 1.161 | 8 |
|  |  | Total | 0.354 | 0.966 | 13 |
|  | Aged | Standard | -0.174 | 0.505 | 6 |
|  |  | Ketogenic | -0.889 | 0.526 | 4 |
|  |  | Total | -0.460 | 0.608 | 10 |
|  | Total | Standard | 0.035 | 0.598 | 11 |
|  |  | Ketogenic | -0.032 | 1.155 | 12 |
|  |  | Total | 0.000 | 0.912 | 23 |
| Gabbr1 | Young | Standard | 0.293 | 0.620 | 6 |
|  |  | Ketogenic | 0.297 | 1.258 | 8 |
|  |  | Total | 0.295 | 1.000 | 14 |
|  | Aged | Standard | -0.110 | 0.961 | 6 |
|  |  | Ketogenic | -0.869 | 0.598 | 4 |
|  |  | Total | -0.414 | 0.886 | 10 |
|  | Total | Standard | 0.092 | 0.799 | 12 |
|  |  | Ketogenic | -0.092 | 1.197 | 12 |
|  |  | Total | 0.000 | 1.000 | 24 |
| Gabbr2 | Young | Standard | 0.475 | 0.870 | 6 |
|  |  | Ketogenic | 0.176 | 1.196 | 8 |
|  |  | Total | 0.304 | 1.041 | 14 |
|  | Aged | Standard | -0.114 | 0.825 | 6 |
|  |  | Ketogenic | -0.894 | 0.556 | 4 |
|  |  | Total | -0.426 | 0.802 | 10 |
|  | Total | Standard | 0.180 | 0.865 | 12 |
|  |  | Ketogenic | -0.180 | 1.128 | 12 |
|  |  | Total | 0.000 | 1.000 | 24 |
| Gabra1 | Young | Standard | 0.298 | 0.794 | 6 |
|  |  | Ketogenic | 0.373 | 1.068 | 8 |
|  |  | Total | 0.341 | 0.926 | 14 |
|  | Aged | Standard | -0.426 | 0.924 | 6 |
|  |  | Ketogenic | -0.554 | 1.102 | 4 |
|  |  | Total | -0.477 | 0.940 | 10 |
|  | Total | Standard | -0.064 | 0.904 | 12 |
|  |  | Ketogenic | 0.064 | 1.125 | 12 |
|  |  | Total | 0.000 | 1.000 | 24 |
| Gabra2 | Young | Standard | 0.285 | 0.908 | 6 |
|  |  | Ketogenic | -0.127 | 1.061 | 8 |
|  |  | Total | 0.050 | 0.984 | 14 |
|  | Aged | Standard | 0.287 | 0.997 | 6 |
|  |  | Ketogenic | -0.605 | 1.073 | 4 |
|  |  | Total | -0.069 | 1.072 | 10 |
|  | Total | Standard | 0.286 | 0.909 | 12 |
|  |  | Ketogenic | -0.286 | 1.042 | 12 |
|  |  | Total | 0.000 | 1.000 | 24 |
| Gabra4 | Young | Standard | 0.595 | 0.803 | 6 |
|  |  | Ketogenic | -0.137 | 1.132 | 8 |
|  |  | Total | 0.177 | 1.039 | 14 |
|  | Aged | Standard | 0.020 | 0.934 | 6 |
|  |  | Ketogenic | -0.649 | 0.911 | 4 |
|  |  | Total | -0.247 | 0.938 | 10 |
|  | Total | Standard | 0.308 | 0.883 | 12 |
|  |  | Ketogenic | -0.308 | 1.051 | 12 |
|  |  | Total | 0.000 | 1.000 | 24 |
| Gabra5 | Young | Standard | 0.676 | 0.647 | 6 |
|  |  | Ketogenic | -0.161 | 1.018 | 8 |
|  |  | Total | 0.197 | 0.950 | 14 |
|  | Aged | Standard | -0.007 | 1.042 | 6 |
|  |  | Ketogenic | -0.681 | 1.069 | 4 |
|  |  | Total | -0.276 | 1.051 | 10 |
|  | Total | Standard | 0.334 | 0.900 | 12 |
|  |  | Ketogenic | -0.334 | 1.018 | 12 |
|  |  | Total | 0.000 | 1.000 | 24 |
| Gabrb1 | Young | Standard | 0.112 | 1.005 | 6 |
|  |  | Ketogenic | 0.192 | 1.088 | 8 |
|  |  | Total | 0.157 | 1.014 | 14 |
|  | Aged | Standard | 0.120 | 1.021 | 6 |
|  |  | Ketogenic | -0.732 | 0.786 | 4 |
|  |  | Total | -0.220 | 0.989 | 10 |
|  | Total | Standard | 0.116 | 0.966 | 12 |
|  |  | Ketogenic | -0.116 | 1.062 | 12 |
|  |  | Total | 0.000 | 1.000 | 24 |
| Gabrb3 | Young | Standard | 0.196 | 0.910 | 6 |
|  |  | Ketogenic | 0.137 | 1.011 | 8 |
|  |  | Total | 0.162 | 0.933 | 14 |
|  | Aged | Standard | -0.031 | 0.962 | 6 |
|  |  | Ketogenic | -0.522 | 1.366 | 4 |
|  |  | Total | -0.227 | 1.095 | 10 |
|  | Total | Standard | 0.082 | 0.901 | 12 |
|  |  | Ketogenic | -0.082 | 1.125 | 12 |
|  |  | Total | 0.000 | 1.000 | 24 |
| Gabrg1 | Young | Standard | 0.677 | 0.702 | 6 |
|  |  | Ketogenic | -0.331 | 0.932 | 8 |
|  |  | Total | 0.101 | 0.962 | 14 |
|  | Aged | Standard | 0.334 | 0.683 | 6 |
|  |  | Ketogenic | -0.854 | 1.280 | 4 |
|  |  | Total | -0.141 | 1.087 | 10 |
|  | Total | Standard | 0.505 | 0.684 | 12 |
|  |  | Ketogenic | -0.505 | 1.032 | 12 |
|  |  | Total | 0.000 | 1.000 | 24 |
| Gabrg2 | Young | Standard | 0.621 | 0.825 | 6 |
|  |  | Ketogenic | -0.005 | 1.134 | 8 |
|  |  | Total | 0.263 | 1.028 | 14 |
|  | Aged | Standard | -0.144 | 0.840 | 6 |
|  |  | Ketogenic | -0.705 | 0.940 | 4 |
|  |  | Total | -0.369 | 0.877 | 10 |
|  | Total | Standard | 0.238 | 0.888 | 12 |
|  |  | Ketogenic | -0.238 | 1.085 | 12 |
|  |  | Total | 0.000 | 1.000 | 24 |
| Gabrg3 | Young | Standard | 0.320 | 0.729 | 6 |
|  |  | Ketogenic | 0.051 | 1.159 | 8 |
|  |  | Total | 0.166 | 0.973 | 14 |
|  | Aged | Standard | -0.285 | 0.638 | 6 |
|  |  | Ketogenic | -0.206 | 0.411 | 3 |
|  |  | Total | -0.259 | 0.546 | 9 |
|  | Total | Standard | 0.018 | 0.725 | 12 |
|  |  | Ketogenic | -0.019 | 0.994 | 11 |
|  |  | Total | 0.000 | 0.844 | 23 |
| Gad1 | Young | Standard | 0.510 | 0.898 | 6 |
|  |  | Ketogenic | 0.096 | 1.243 | 8 |
|  |  | Total | 0.274 | 1.089 | 14 |
|  | Aged | Standard | -0.245 | 0.798 | 6 |
|  |  | Ketogenic | -0.590 | 0.728 | 4 |
|  |  | Total | -0.383 | 0.750 | 10 |
|  | Total | Standard | 0.133 | 0.901 | 12 |
|  |  | Ketogenic | -0.133 | 1.114 | 12 |
|  |  | Total | 0.000 | 1.000 | 24 |
| Gls | Young | Standard | 0.562 | 0.921 | 6 |
|  |  | Ketogenic | -0.225 | 1.150 | 8 |
|  |  | Total | 0.112 | 1.096 | 14 |
|  | Aged | Standard | 0.075 | 0.900 | 6 |
|  |  | Ketogenic | -0.505 | 0.836 | 4 |
|  |  | Total | -0.157 | 0.879 | 10 |
|  | Total | Standard | 0.318 | 0.905 | 12 |
|  |  | Ketogenic | -0.318 | 1.026 | 12 |
|  |  | Total | 0.000 | 1.000 | 24 |
| Glul | Young | Standard | -0.338 | 1.052 | 6 |
|  |  | Ketogenic | 0.241 | 0.716 | 8 |
|  |  | Total | -0.008 | 0.889 | 14 |
|  | Aged | Standard | 0.219 | 1.355 | 6 |
|  |  | Ketogenic | -0.302 | 0.983 | 4 |
|  |  | Total | 0.011 | 1.189 | 10 |
|  | Total | Standard | -0.060 | 1.192 | 12 |
|  |  | Ketogenic | 0.060 | 0.813 | 12 |
|  |  | Total | 0.000 | 1.000 | 24 |
| Gnai1 | Young | Standard | 0.418 | 0.858 | 6 |
|  |  | Ketogenic | -0.072 | 1.122 | 8 |
|  |  | Total | 0.138 | 1.012 | 14 |
|  | Aged | Standard | 0.107 | 0.924 | 6 |
|  |  | Ketogenic | -0.643 | 1.070 | 4 |
|  |  | Total | -0.193 | 1.003 | 10 |
|  | Total | Standard | 0.262 | 0.865 | 12 |
|  |  | Ketogenic | -0.262 | 1.092 | 12 |
|  |  | Total | 0.000 | 1.000 | 24 |
| Gnaq | Young | Standard | 0.561 | 0.921 | 6 |
|  |  | Ketogenic | -0.136 | 1.025 | 8 |
|  |  | Total | 0.163 | 1.010 | 14 |
|  | Aged | Standard | 0.075 | 0.916 | 6 |
|  |  | Ketogenic | -0.682 | 1.044 | 4 |
|  |  | Total | -0.228 | 0.991 | 10 |
|  | Total | Standard | 0.318 | 0.912 | 12 |
|  |  | Ketogenic | -0.318 | 1.019 | 12 |
|  |  | Total | 0.000 | 1.000 | 24 |
| Gphn | Young | Standard | 0.596 | 0.955 | 6 |
|  |  | Ketogenic | -0.167 | 1.035 | 8 |
|  |  | Total | 0.160 | 1.040 | 14 |
|  | Aged | Standard | 0.042 | 0.888 | 6 |
|  |  | Ketogenic | -0.622 | 1.016 | 4 |
|  |  | Total | -0.224 | 0.948 | 10 |
|  | Total | Standard | 0.319 | 0.925 | 12 |
|  |  | Ketogenic | -0.319 | 1.006 | 12 |
|  |  | Total | 0.000 | 1.000 | 24 |
| Gria1 | Young | Standard | 0.716 | 0.798 | 6 |
|  |  | Ketogenic | 0.155 | 1.090 | 8 |
|  |  | Total | 0.396 | 0.983 | 14 |
|  | Aged | Standard | -0.318 | 0.682 | 6 |
|  |  | Ketogenic | -0.907 | 0.819 | 4 |
|  |  | Total | -0.554 | 0.758 | 10 |
|  | Total | Standard | 0.199 | 0.890 | 12 |
|  |  | Ketogenic | -0.199 | 1.101 | 12 |
|  |  | Total | 0.000 | 1.000 | 24 |
| Gria2 | Young | Standard | 0.793 | 0.735 | 6 |
|  |  | Ketogenic | -0.068 | 1.134 | 8 |
|  |  | Total | 0.301 | 1.047 | 14 |
|  | Aged | Standard | -0.222 | 0.739 | 6 |
|  |  | Ketogenic | -0.721 | 0.892 | 4 |
|  |  | Total | -0.422 | 0.797 | 10 |
|  | Total | Standard | 0.285 | 0.880 | 12 |
|  |  | Ketogenic | -0.285 | 1.067 | 12 |
|  |  | Total | 0.000 | 1.000 | 24 |
| Gria3 | Young | Standard | 0.751 | 0.654 | 6 |
|  |  | Ketogenic | -0.101 | 1.099 | 8 |
|  |  | Total | 0.264 | 1.003 | 14 |
|  | Aged | Standard | -0.055 | 0.819 | 6 |
|  |  | Ketogenic | -0.842 | 0.954 | 4 |
|  |  | Total | -0.370 | 0.917 | 10 |
|  | Total | Standard | 0.348 | 0.823 | 12 |
|  |  | Ketogenic | -0.348 | 1.072 | 12 |
|  |  | Total | 0.000 | 1.000 | 24 |
| Gria4 | Young | Standard | 0.782 | 0.824 | 6 |
|  |  | Ketogenic | -0.123 | 1.112 | 8 |
|  |  | Total | 0.265 | 1.069 | 14 |
|  | Aged | Standard | -0.108 | 0.596 | 6 |
|  |  | Ketogenic | -0.766 | 0.995 | 4 |
|  |  | Total | -0.371 | 0.802 | 10 |
|  | Total | Standard | 0.337 | 0.828 | 12 |
|  |  | Ketogenic | -0.337 | 1.076 | 12 |
|  |  | Total | 0.000 | 1.000 | 24 |
| Grik1 | Young | Standard | 0.542 | 0.660 | 6 |
|  |  | Ketogenic | -0.143 | 1.108 | 8 |
|  |  | Total | 0.151 | 0.976 | 14 |
|  | Aged | Standard | 0.097 | 1.031 | 6 |
|  |  | Ketogenic | -0.673 | 1.020 | 4 |
|  |  | Total | -0.211 | 1.047 | 10 |
|  | Total | Standard | 0.319 | 0.857 | 12 |
|  |  | Ketogenic | -0.319 | 1.065 | 12 |
|  |  | Total | 0.000 | 1.000 | 24 |
| Grik2 | Young | Standard | 0.537 | 0.843 | 6 |
|  |  | Ketogenic | -0.085 | 1.204 | 8 |
|  |  | Total | 0.182 | 1.075 | 14 |
|  | Aged | Standard | 0.030 | 0.862 | 6 |
|  |  | Ketogenic | -0.682 | 0.803 | 4 |
|  |  | Total | -0.255 | 0.874 | 10 |
|  | Total | Standard | 0.284 | 0.855 | 12 |
|  |  | Ketogenic | -0.284 | 1.088 | 12 |
|  |  | Total | 0.000 | 1.000 | 24 |
| Grik5 | Young | Standard | 0.335 | 0.783 | 6 |
|  |  | Ketogenic | 0.161 | 1.314 | 8 |
|  |  | Total | 0.236 | 1.083 | 14 |
|  | Aged | Standard | -0.082 | 0.862 | 6 |
|  |  | Ketogenic | -0.702 | 0.642 | 4 |
|  |  | Total | -0.330 | 0.808 | 10 |
|  | Total | Standard | 0.127 | 0.815 | 12 |
|  |  | Ketogenic | -0.127 | 1.180 | 12 |
|  |  | Total | 0.000 | 1.000 | 24 |
| Grin1 | Young | Standard | 0.342 | 1.024 | 6 |
|  |  | Ketogenic | 0.067 | 1.122 | 8 |
|  |  | Total | 0.184 | 1.049 | 14 |
|  | Aged | Standard | -0.113 | 1.066 | 6 |
|  |  | Ketogenic | -0.476 | 0.718 | 4 |
|  |  | Total | -0.258 | 0.916 | 10 |
|  | Total | Standard | 0.114 | 1.025 | 12 |
|  |  | Ketogenic | -0.114 | 1.006 | 12 |
|  |  | Total | 0.000 | 1.000 | 24 |
| Grin2a | Young | Standard | 0.511 | 0.802 | 6 |
|  |  | Ketogenic | -0.003 | 1.037 | 8 |
|  |  | Total | 0.217 | 0.947 | 14 |
|  | Aged | Standard | -0.004 | 0.906 | 6 |
|  |  | Ketogenic | -0.756 | 1.196 | 4 |
|  |  | Total | -0.304 | 1.041 | 10 |
|  | Total | Standard | 0.254 | 0.859 | 12 |
|  |  | Ketogenic | -0.254 | 1.101 | 12 |
|  |  | Total | 0.000 | 1.000 | 24 |
| Grin2b | Young | Standard | 0.506 | 0.685 | 6 |
|  |  | Ketogenic | 0.016 | 1.210 | 8 |
|  |  | Total | 0.226 | 1.016 | 14 |
|  | Aged | Standard | -0.053 | 0.881 | 6 |
|  |  | Ketogenic | -0.713 | 0.988 | 4 |
|  |  | Total | -0.317 | 0.934 | 10 |
|  | Total | Standard | 0.227 | 0.807 | 12 |
|  |  | Ketogenic | -0.227 | 1.152 | 12 |
|  |  | Total | 0.000 | 1.000 | 24 |
| Grm3 | Young | Standard | 0.569 | 0.881 | 6 |
|  |  | Ketogenic | -0.300 | 1.027 | 8 |
|  |  | Total | 0.072 | 1.032 | 14 |
|  | Aged | Standard | 0.268 | 0.883 | 6 |
|  |  | Ketogenic | -0.655 | 1.006 | 4 |
|  |  | Total | -0.101 | 0.999 | 10 |
|  | Total | Standard | 0.418 | 0.856 | 12 |
|  |  | Ketogenic | -0.418 | 0.988 | 12 |
|  |  | Total | 0.000 | 1.000 | 24 |
| Grm5 | Young | Standard | 0.645 | 0.682 | 6 |
|  |  | Ketogenic | 0.132 | 1.259 | 8 |
|  |  | Total | 0.352 | 1.050 | 14 |
|  | Aged | Standard | -0.261 | 0.773 | 6 |
|  |  | Ketogenic | -0.840 | 0.491 | 4 |
|  |  | Total | -0.493 | 0.709 | 10 |
|  | Total | Standard | 0.192 | 0.841 | 12 |
|  |  | Ketogenic | -0.192 | 1.142 | 12 |
|  |  | Total | 0.000 | 1.000 | 24 |
| Grm7 | Young | Standard | 0.562 | 0.873 | 6 |
|  |  | Ketogenic | -0.094 | 1.168 | 8 |
|  |  | Total | 0.187 | 1.068 | 14 |
|  | Aged | Standard | 0.120 | 0.781 | 6 |
|  |  | Ketogenic | -0.835 | 0.765 | 4 |
|  |  | Total | -0.262 | 0.881 | 10 |
|  | Total | Standard | 0.341 | 0.823 | 12 |
|  |  | Ketogenic | -0.341 | 1.077 | 12 |
|  |  | Total | 0.000 | 1.000 | 24 |
| Homer1 | Young | Standard | 0.679 | 0.930 | 6 |
|  |  | Ketogenic | -0.150 | 1.104 | 8 |
|  |  | Total | 0.205 | 1.082 | 14 |
|  | Aged | Standard | -0.064 | 0.808 | 6 |
|  |  | Ketogenic | -0.624 | 0.886 | 4 |
|  |  | Total | -0.288 | 0.841 | 10 |
|  | Total | Standard | 0.308 | 0.916 | 12 |
|  |  | Ketogenic | -0.308 | 1.022 | 12 |
|  |  | Total | 0.000 | 1.000 | 24 |
| Itpr1 | Young | Standard | 0.505 | 0.778 | 6 |
|  |  | Ketogenic | -0.139 | 1.046 | 8 |
|  |  | Total | 0.137 | 0.965 | 14 |
|  | Aged | Standard | 0.094 | 1.131 | 6 |
|  |  | Ketogenic | -0.621 | 0.939 | 4 |
|  |  | Total | -0.192 | 1.068 | 10 |
|  | Total | Standard | 0.300 | 0.950 | 12 |
|  |  | Ketogenic | -0.300 | 0.996 | 12 |
|  |  | Total | 0.000 | 1.000 | 24 |
| Mapk1 | Young | Standard | 0.667 | 0.835 | 6 |
|  |  | Ketogenic | 0.139 | 1.028 | 8 |
|  |  | Total | 0.365 | 0.954 | 14 |
|  | Aged | Standard | -0.470 | 0.959 | 6 |
|  |  | Ketogenic | -0.573 | 0.828 | 4 |
|  |  | Total | -0.511 | 0.862 | 10 |
|  | Total | Standard | 0.098 | 1.043 | 12 |
|  |  | Ketogenic | -0.098 | 0.991 | 12 |
|  |  | Total | 0.000 | 1.000 | 24 |
| Nsf | Young | Standard | 0.776 | 0.741 | 6 |
|  |  | Ketogenic | 0.116 | 1.218 | 8 |
|  |  | Total | 0.399 | 1.060 | 14 |
|  | Aged | Standard | -0.365 | 0.608 | 6 |
|  |  | Ketogenic | -0.850 | 0.454 | 4 |
|  |  | Total | -0.559 | 0.580 | 10 |
|  | Total | Standard | 0.206 | 0.879 | 12 |
|  |  | Ketogenic | -0.206 | 1.107 | 12 |
|  |  | Total | 0.000 | 1.000 | 24 |
| P2rx7 | Young | Standard | 0.289 | 0.970 | 6 |
|  |  | Ketogenic | -0.058 | 0.806 | 7 |
|  |  | Total | 0.102 | 0.866 | 13 |
|  | Aged | Standard | 0.066 | 0.478 | 6 |
|  |  | Ketogenic | -0.576 | 0.971 | 3 |
|  |  | Total | -0.148 | 0.694 | 9 |
|  | Total | Standard | 0.178 | 0.738 | 12 |
|  |  | Ketogenic | -0.213 | 0.840 | 10 |
|  |  | Total | 0.000 | 0.792 | 22 |
| Phgdh | Young | Standard | 0.733 | 0.998 | 6 |
|  |  | Ketogenic | -0.179 | 1.140 | 8 |
|  |  | Total | 0.211 | 1.141 | 14 |
|  | Aged | Standard | -0.026 | 0.554 | 6 |
|  |  | Ketogenic | -0.700 | 0.804 | 4 |
|  |  | Total | -0.296 | 0.712 | 10 |
|  | Total | Standard | 0.353 | 0.866 | 12 |
|  |  | Ketogenic | -0.353 | 1.034 | 12 |
|  |  | Total | 0.000 | 1.000 | 24 |
| Pla2g6 | Young | Standard | 0.546 | 0.718 | 6 |
|  |  | Ketogenic | -0.015 | 0.994 | 8 |
|  |  | Total | 0.226 | 0.902 | 14 |
|  | Aged | Standard | -0.024 | 0.611 | 6 |
|  |  | Ketogenic | -1.006 | 0.506 | 3 |
|  |  | Total | -0.351 | 0.734 | 9 |
|  | Total | Standard | 0.261 | 0.702 | 12 |
|  |  | Ketogenic | -0.285 | 0.979 | 11 |
|  |  | Total | 0.000 | 0.871 | 23 |
| Plcb1 | Young | Standard | 0.725 | 0.776 | 6 |
|  |  | Ketogenic | -0.138 | 1.048 | 8 |
|  |  | Total | 0.232 | 1.010 | 14 |
|  | Aged | Standard | -0.090 | 0.953 | 6 |
|  |  | Ketogenic | -0.677 | 0.924 | 4 |
|  |  | Total | -0.325 | 0.938 | 10 |
|  | Total | Standard | 0.318 | 0.932 | 12 |
|  |  | Ketogenic | -0.318 | 1.001 | 12 |
|  |  | Total | 0.000 | 1.000 | 24 |
| Prodh | Young | Standard | 0.080 | 0.658 | 6 |
|  |  | Ketogenic | 0.070 | 1.069 | 8 |
|  |  | Total | 0.074 | 0.884 | 14 |
|  | Aged | Standard | 0.200 | 1.433 | 6 |
|  |  | Ketogenic | -0.560 | 0.573 | 4 |
|  |  | Total | -0.104 | 1.185 | 10 |
|  | Total | Standard | 0.140 | 1.065 | 12 |
|  |  | Ketogenic | -0.140 | 0.956 | 12 |
|  |  | Total | 0.000 | 1.000 | 24 |
| Shank2 | Young | Standard | 0.366 | 0.771 | 6 |
|  |  | Ketogenic | 0.018 | 1.335 | 8 |
|  |  | Total | 0.167 | 1.104 | 14 |
|  | Aged | Standard | 0.006 | 0.892 | 6 |
|  |  | Ketogenic | -0.594 | 0.674 | 4 |
|  |  | Total | -0.234 | 0.831 | 10 |
|  | Total | Standard | 0.186 | 0.817 | 12 |
|  |  | Ketogenic | -0.186 | 1.161 | 12 |
|  |  | Total | 0.000 | 1.000 | 24 |
| Slc17a7 | Young | Standard | 0.275 | 0.736 | 6 |
|  |  | Ketogenic | 0.220 | 1.251 | 8 |
|  |  | Total | 0.244 | 1.026 | 14 |
|  | Aged | Standard | -0.089 | 1.044 | 6 |
|  |  | Ketogenic | -0.720 | 0.555 | 4 |
|  |  | Total | -0.341 | 0.902 | 10 |
|  | Total | Standard | 0.093 | 0.882 | 12 |
|  |  | Ketogenic | -0.093 | 1.138 | 12 |
|  |  | Total | 0.000 | 1.000 | 24 |
| Slc1a1 | Young | Standard | 0.430 | 0.809 | 6 |
|  |  | Ketogenic | -0.039 | 0.965 | 8 |
|  |  | Total | 0.162 | 0.901 | 14 |
|  | Aged | Standard | 0.115 | 1.142 | 6 |
|  |  | Ketogenic | -0.739 | 1.048 | 4 |
|  |  | Total | -0.227 | 1.134 | 10 |
|  | Total | Standard | 0.272 | 0.958 | 12 |
|  |  | Ketogenic | -0.272 | 1.006 | 12 |
|  |  | Total | 0.000 | 1.000 | 24 |
| Slc1a2 | Young | Standard | 0.404 | 0.776 | 6 |
|  |  | Ketogenic | -0.025 | 1.133 | 8 |
|  |  | Total | 0.159 | 0.985 | 14 |
|  | Aged | Standard | 0.299 | 0.935 | 6 |
|  |  | Ketogenic | -1.004 | 0.599 | 4 |
|  |  | Total | -0.222 | 1.029 | 10 |
|  | Total | Standard | 0.352 | 0.821 | 12 |
|  |  | Ketogenic | -0.352 | 1.071 | 12 |
|  |  | Total | 0.000 | 1.000 | 24 |
| Slc1a3 | Young | Standard | 0.821 | 0.848 | 6 |
|  |  | Ketogenic | -0.320 | 1.093 | 8 |
|  |  | Total | 0.169 | 1.124 | 14 |
|  | Aged | Standard | 0.113 | 0.648 | 6 |
|  |  | Ketogenic | -0.761 | 0.751 | 4 |
|  |  | Total | -0.237 | 0.790 | 10 |
|  | Total | Standard | 0.467 | 0.809 | 12 |
|  |  | Ketogenic | -0.467 | 0.981 | 12 |
|  |  | Total | 0.000 | 1.000 | 24 |
| Slc38a1 | Young | Standard | 0.636 | 0.602 | 6 |
|  |  | Ketogenic | -0.100 | 1.286 | 8 |
|  |  | Total | 0.215 | 1.083 | 14 |
|  | Aged | Standard | 0.154 | 0.624 | 6 |
|  |  | Ketogenic | -0.985 | 0.617 | 4 |
|  |  | Total | -0.301 | 0.830 | 10 |
|  | Total | Standard | 0.395 | 0.636 | 12 |
|  |  | Ketogenic | -0.395 | 1.160 | 12 |
|  |  | Total | 0.000 | 1.000 | 24 |
| Slc6a1 | Young | Standard | 0.654 | 0.642 | 6 |
|  |  | Ketogenic | -0.012 | 1.267 | 8 |
|  |  | Total | 0.274 | 1.068 | 14 |
|  | Aged | Standard | 0.034 | 0.678 | 6 |
|  |  | Ketogenic | -1.008 | 0.503 | 4 |
|  |  | Total | -0.383 | 0.793 | 10 |
|  | Total | Standard | 0.344 | 0.708 | 12 |
|  |  | Ketogenic | -0.344 | 1.154 | 12 |
|  |  | Total | 0.000 | 1.000 | 24 |
| Slc6a11 | Young | Standard | 0.397 | 0.744 | 6 |
|  |  | Ketogenic | -0.044 | 1.186 | 8 |
|  |  | Total | 0.145 | 1.011 | 14 |
|  | Aged | Standard | 0.239 | 1.038 | 6 |
|  |  | Ketogenic | -0.865 | 0.484 | 4 |
|  |  | Total | -0.203 | 1.001 | 10 |
|  | Total | Standard | 0.318 | 0.865 | 12 |
|  |  | Ketogenic | -0.318 | 1.060 | 12 |
|  |  | Total | 0.000 | 1.000 | 24 |
| Snca | Young | Standard | 0.397 | 0.974 | 6 |
|  |  | Ketogenic | 0.075 | 0.948 | 8 |
|  |  | Total | 0.213 | 0.936 | 14 |
|  | Aged | Standard | 0.015 | 1.207 | 6 |
|  |  | Ketogenic | -0.766 | 0.669 | 4 |
|  |  | Total | -0.298 | 1.059 | 10 |
|  | Total | Standard | 0.206 | 1.064 | 12 |
|  |  | Ketogenic | -0.206 | 0.931 | 12 |
|  |  | Total | 0.000 | 1.000 | 24 |
| Srr | Young | Standard | 0.440 | 0.854 | 6 |
|  |  | Ketogenic | -0.161 | 0.843 | 8 |
|  |  | Total | 0.096 | 0.871 | 14 |
|  | Aged | Standard | 0.182 | 1.330 | 6 |
|  |  | Ketogenic | -0.610 | 0.907 | 4 |
|  |  | Total | -0.135 | 1.194 | 10 |
|  | Total | Standard | 0.311 | 1.074 | 12 |
|  |  | Ketogenic | -0.311 | 0.852 | 12 |
|  |  | Total | 0.000 | 1.000 | 24 |

Table S7: Descriptive statistics for gene expression within CA1.

| Effect | Gene | Type III Sum of Squares | df | Mean Square | F | p-values | Partial Eta Squared | Noncent. Parameter | Observed Power^bz^ | Rank | FDR q-values 0.5% |
| --- | --- | --- | --- | --- | --- | --- | --- | --- | --- | --- | --- |
| Age | Abat | 3.273 | 1 | 3.273 | 3.503 | 0.076 | 0.149 | 3.503 | 0.429 | 14 | 0.001 |
|  | Adora1 | 1.998 | 1 | 1.998 | 1.951 | 0.178 | 0.089 | 1.951 | 0.265 | 30 | 0.003 |
|  | Aldh5a1 | 1.004 | 1 | 1.004 | 0.990 | 0.332 | 0.047 | 0.990 | 0.158 | 49 | 0.004 |
|  | App | 2.136 | 1 | 2.136 | 2.126 | 0.160 | 0.096 | 2.126 | 0.284 | 29 | 0.003 |
|  | Cdk5r1 | 1.540 | 1 | 1.540 | 1.491 | 0.236 | 0.069 | 1.491 | 0.214 | 36 | 0.003 |
|  | Dlg4 | 4.106 | 1 | 4.106 | 5.880 | 0.025 | 0.236 | 5.880 | 0.634 | 3 | 0.000 |
|  | Gabbr1 | 3.478 | 1 | 3.478 | 3.723 | 0.068 | 0.157 | 3.723 | 0.451 | 13 | 0.001 |
|  | Gabbr2 | 3.887 | 1 | 3.887 | 4.290 | 0.051 | 0.177 | 4.290 | 0.505 | 9 | 0.001 |
|  | Gabra1 | 3.845 | 1 | 3.845 | 4.039 | 0.058 | 0.168 | 4.039 | 0.481 | 11 | 0.001 |
|  | Gabra2 | 0.319 | 1 | 0.319 | 0.312 | 0.582 | 0.015 | 0.312 | 0.083 | 54 | 0.005 |
|  | Gabra4 | 1.668 | 1 | 1.668 | 1.752 | 0.201 | 0.081 | 1.752 | 0.243 | 33 | 0.003 |
|  | Gabra5 | 2.039 | 1 | 2.039 | 2.241 | 0.150 | 0.101 | 2.241 | 0.297 | 26 | 0.002 |
|  | Gabrb1 | 1.181 | 1 | 1.181 | 1.157 | 0.295 | 0.055 | 1.157 | 0.176 | 44 | 0.004 |
|  | Gabrb3 | 1.107 | 1 | 1.107 | 1.029 | 0.323 | 0.049 | 1.029 | 0.162 | 48 | 0.004 |
|  | Gabrg1 | 1.059 | 1 | 1.059 | 1.341 | 0.260 | 0.063 | 1.341 | 0.197 | 40 | 0.004 |
|  | Gabrg2 | 3.033 | 1 | 3.033 | 3.266 | 0.086 | 0.140 | 3.266 | 0.405 | 15 | 0.001 |
|  | Gabrg3 | 0.939 | 1 | 0.939 | 1.236 | 0.280 | 0.061 | 1.236 | 0.184 | 43 | 0.004 |
|  | Gad1 | 2.932 | 1 | 2.932 | 2.990 | 0.099 | 0.130 | 2.990 | 0.377 | 17 | 0.002 |
|  | Gls | 0.831 | 1 | 0.831 | 0.846 | 0.369 | 0.041 | 0.846 | 0.142 | 50 | 0.005 |
|  | Glul | 0.000 | 1 | 0.000 | 0.000 | 0.986 | 0.000 | 0.000 | 0.050 | 55 | 0.005 |
|  | Gnai1 | 1.098 | 1 | 1.098 | 1.088 | 0.309 | 0.052 | 1.088 | 0.168 | 46 | 0.004 |
|  | Gnaq | 1.506 | 1 | 1.506 | 1.580 | 0.223 | 0.073 | 1.580 | 0.224 | 34 | 0.003 |
|  | Gphn | 1.436 | 1 | 1.436 | 1.504 | 0.234 | 0.070 | 1.504 | 0.215 | 35 | 0.003 |
|  | Gria1 | 6.206 | 1 | 6.206 | 7.839 | 0.011 | 0.282 | 7.839 | 0.759 | 2 | 0.000 |
|  | Gria2 | 3.929 | 1 | 3.929 | 4.673 | 0.043 | 0.189 | 4.673 | 0.539 | 7 | 0.001 |
|  | Gria3 | 3.382 | 1 | 3.382 | 4.057 | 0.058 | 0.169 | 4.057 | 0.483 | 10 | 0.001 |
|  | Gria4 | 3.318 | 1 | 3.318 | 3.952 | 0.061 | 0.165 | 3.952 | 0.473 | 12 | 0.001 |
|  | Grik1 | 1.343 | 1 | 1.343 | 1.399 | 0.251 | 0.065 | 1.399 | 0.203 | 39 | 0.004 |
|  | Grik2 | 1.723 | 1 | 1.723 | 1.782 | 0.197 | 0.082 | 1.782 | 0.246 | 32 | 0.003 |
|  | Grik5 | 2.317 | 1 | 2.317 | 2.306 | 0.145 | 0.103 | 2.306 | 0.304 | 24 | 0.002 |
|  | Grin1 | 1.404 | 1 | 1.404 | 1.319 | 0.264 | 0.062 | 1.319 | 0.194 | 41 | 0.004 |
|  | Grin2a | 2.268 | 1 | 2.268 | 2.369 | 0.139 | 0.106 | 2.369 | 0.311 | 23 | 0.002 |
|  | Grin2b | 2.341 | 1 | 2.341 | 2.412 | 0.136 | 0.108 | 2.412 | 0.315 | 20 | 0.002 |
|  | Grm3 | 0.607 | 1 | 0.607 | 0.667 | 0.424 | 0.032 | 0.667 | 0.122 | 52 | 0.005 |
|  | Grm5 | 4.981 | 1 | 4.981 | 5.815 | 0.026 | 0.225 | 5.815 | 0.631 | 4 | 0.000 |
|  | Grm7 | 1.978 | 1 | 1.978 | 2.179 | 0.155 | 0.098 | 2.179 | 0.290 | 27 | 0.002 |
|  | Homer1 | 2.089 | 1 | 2.089 | 2.262 | 0.148 | 0.102 | 2.262 | 0.299 | 25 | 0.002 |
|  | Itpr1 | 1.124 | 1 | 1.124 | 1.140 | 0.298 | 0.054 | 1.140 | 0.174 | 45 | 0.004 |
|  | Mapk1 | 4.824 | 1 | 4.824 | 5.500 | 0.029 | 0.216 | 5.500 | 0.607 | 5 | 0.000 |
|  | Nsf | 6.267 | 1 | 6.267 | 8.040 | 0.010 | 0.287 | 8.040 | 0.770 | 1 | 0.000 |
|  | P2rx7 | 0.678 | 1 | 0.678 | 1.049 | 0.319 | 0.055 | 1.049 | 0.163 | 47 | 0.004 |
|  | Phgdh | 2.312 | 1 | 2.312 | 2.634 | 0.120 | 0.116 | 2.634 | 0.339 | 19 | 0.002 |
|  | Pla2g6 | 3.079 | 1 | 3.079 | 4.926 | 0.039 | 0.206 | 4.926 | 0.558 | 6 | 0.001 |
|  | Plcb1 | 2.593 | 1 | 2.593 | 2.912 | 0.103 | 0.127 | 2.912 | 0.369 | 18 | 0.002 |
|  | Prodh | 0.368 | 1 | 0.368 | 0.344 | 0.564 | 0.017 | 0.344 | 0.086 | 53 | 0.005 |
|  | Shank2 | 1.331 | 1 | 1.331 | 1.281 | 0.271 | 0.060 | 1.281 | 0.190 | 42 | 0.004 |
|  | Slc17a7 | 2.398 | 1 | 2.398 | 2.393 | 0.138 | 0.107 | 2.393 | 0.313 | 21 | 0.002 |
|  | Slc1a1 | 1.454 | 1 | 1.454 | 1.483 | 0.238 | 0.069 | 1.483 | 0.213 | 37 | 0.003 |
|  | Slc1a2 | 1.657 | 1 | 1.657 | 1.900 | 0.183 | 0.087 | 1.900 | 0.259 | 31 | 0.003 |
|  | Slc1a3 | 1.866 | 1 | 1.866 | 2.371 | 0.139 | 0.106 | 2.371 | 0.311 | 22 | 0.002 |
|  | Slc38a1 | 2.633 | 1 | 2.633 | 3.197 | 0.089 | 0.138 | 3.197 | 0.398 | 16 | 0.001 |
|  | Slc6a1 | 3.688 | 1 | 3.688 | 4.507 | 0.046 | 0.184 | 4.507 | 0.524 | 8 | 0.001 |
|  | Slc6a11 | 1.354 | 1 | 1.354 | 1.448 | 0.243 | 0.068 | 1.448 | 0.209 | 38 | 0.003 |
|  | Snca | 2.112 | 1 | 2.112 | 2.148 | 0.158 | 0.097 | 2.148 | 0.287 | 28 | 0.003 |
|  | Srr | 0.706 | 1 | 0.706 | 0.708 | 0.410 | 0.034 | 0.708 | 0.126 | 51 | 0.005 |
| Diet | Abat | 1.732 | 1 | 1.732 | 1.853 | 0.189 | 0.085 | 1.853 | 0.254 | 36 | 0.003 |
|  | Adora1 | 0.392 | 1 | 0.392 | 0.383 | 0.543 | 0.019 | 0.383 | 0.091 | 52 | 0.005 |
|  | Aldh5a1 | 2.079 | 1 | 2.079 | 2.050 | 0.168 | 0.093 | 2.050 | 0.276 | 32 | 0.003 |
|  | App | 1.082 | 1 | 1.082 | 1.076 | 0.312 | 0.051 | 1.076 | 0.167 | 40 | 0.004 |
|  | Cdk5r1 | 0.221 | 1 | 0.221 | 0.196 | 0.663 | 0.010 | 0.196 | 0.071 | 41 | 0.004 |
|  | Dlg4 | 0.583 | 1 | 0.583 | 0.523 | 0.478 | 0.025 | 0.523 | 0.106 | 47 | 0.004 |
|  | Gabbr1 | 0.805 | 1 | 0.805 | 0.862 | 0.364 | 0.041 | 0.862 | 0.143 | 43 | 0.004 |
|  | Gabbr2 | 1.642 | 1 | 1.642 | 1.812 | 0.193 | 0.083 | 1.812 | 0.250 | 37 | 0.003 |
|  | Gabra1 | 0.004 | 1 | 0.004 | 0.004 | 0.950 | 0.000 | 0.004 | 0.050 | 55 | 0.005 |
|  | Gabra2 | 2.403 | 1 | 2.403 | 2.353 | 0.141 | 0.105 | 2.353 | 0.309 | 29 | 0.003 |
|  | Gabra4 | 2.772 | 1 | 2.772 | 2.912 | 0.103 | 0.127 | 2.912 | 0.369 | 20 | 0.002 |
|  | Gabra5 | 3.222 | 1 | 3.222 | 3.541 | 0.075 | 0.150 | 3.541 | 0.433 | 13 | 0.001 |
|  | Gabrb1 | 0.842 | 1 | 0.842 | 0.826 | 0.374 | 0.040 | 0.826 | 0.139 | 45 | 0.004 |
|  | Gabrb3 | 0.426 | 1 | 0.426 | 0.396 | 0.536 | 0.019 | 0.396 | 0.092 | 51 | 0.005 |
|  | Gabrg1 | 6.811 | 1 | 6.811 | 8.629 | 0.008 | 0.301 | 8.629 | 0.798 | 1 | 0.000 |
|  | Gabrg2 | 1.988 | 1 | 1.988 | 2.141 | 0.159 | 0.097 | 2.141 | 0.286 | 31 | 0.003 |
|  | Gabrg3 | 0.046 | 1 | 0.046 | 0.060 | 0.808 | 0.003 | 0.060 | 0.056 | 53 | 0.005 |
|  | Gad1 | 0.813 | 1 | 0.813 | 0.829 | 0.373 | 0.040 | 0.829 | 0.140 | 44 | 0.004 |
|  | Gls | 2.635 | 1 | 2.635 | 2.682 | 0.117 | 0.118 | 2.682 | 0.345 | 22 | 0.002 |
|  | Glul | 0.005 | 1 | 0.005 | 0.005 | 0.947 | 0.000 | 0.005 | 0.050 | 54 | 0.005 |
|  | Gnai1 | 2.169 | 1 | 2.169 | 2.148 | 0.158 | 0.097 | 2.148 | 0.287 | 30 | 0.003 |
|  | Gnaq | 2.984 | 1 | 2.984 | 3.130 | 0.092 | 0.135 | 3.130 | 0.391 | 15 | 0.001 |
|  | Gphn | 2.877 | 1 | 2.877 | 3.015 | 0.098 | 0.131 | 3.015 | 0.380 | 18 | 0.002 |
|  | Gria1 | 1.865 | 1 | 1.865 | 2.356 | 0.141 | 0.105 | 2.356 | 0.309 | 28 | 0.003 |
|  | Gria2 | 2.609 | 1 | 2.609 | 3.103 | 0.093 | 0.134 | 3.103 | 0.389 | 17 | 0.002 |
|  | Gria3 | 3.797 | 1 | 3.797 | 4.554 | 0.045 | 0.185 | 4.554 | 0.528 | 8 | 0.001 |
|  | Gria4 | 3.452 | 1 | 3.452 | 4.111 | 0.056 | 0.171 | 4.111 | 0.488 | 9 | 0.001 |
|  | Grik1 | 2.989 | 1 | 2.989 | 3.112 | 0.093 | 0.135 | 3.112 | 0.390 | 16 | 0.001 |
|  | Grik2 | 2.513 | 1 | 2.513 | 2.598 | 0.123 | 0.115 | 2.598 | 0.336 | 24 | 0.002 |
|  | Grik5 | 0.891 | 1 | 0.891 | 0.886 | 0.358 | 0.042 | 0.886 | 0.146 | 42 | 0.004 |
|  | Grin1 | 0.575 | 1 | 0.575 | 0.540 | 0.471 | 0.026 | 0.540 | 0.108 | 50 | 0.005 |
|  | Grin2a | 2.263 | 1 | 2.263 | 2.364 | 0.140 | 0.106 | 2.364 | 0.310 | 27 | 0.002 |
|  | Grin2b | 1.867 | 1 | 1.867 | 1.923 | 0.181 | 0.088 | 1.923 | 0.262 | 34 | 0.003 |
|  | Grm3 | 4.530 | 1 | 4.530 | 4.979 | 0.037 | 0.199 | 4.979 | 0.565 | 5 | 0.000 |
|  | Grm5 | 1.684 | 1 | 1.684 | 1.966 | 0.176 | 0.090 | 1.966 | 0.267 | 33 | 0.003 |
|  | Grm7 | 3.665 | 1 | 3.665 | 4.038 | 0.058 | 0.168 | 4.038 | 0.481 | 11 | 0.001 |
|  | Homer1 | 2.723 | 1 | 2.723 | 2.948 | 0.101 | 0.128 | 2.948 | 0.373 | 19 | 0.002 |
|  | Itpr1 | 2.609 | 1 | 2.609 | 2.646 | 0.119 | 0.117 | 2.646 | 0.341 | 23 | 0.002 |
|  | Mapk1 | 0.562 | 1 | 0.562 | 0.641 | 0.433 | 0.031 | 0.641 | 0.119 | 49 | 0.004 |
|  | Nsf | 1.850 | 1 | 1.850 | 2.374 | 0.139 | 0.106 | 2.374 | 0.311 | 26 | 0.002 |
|  | P2rx7 | 1.208 | 1 | 1.208 | 1.869 | 0.188 | 0.094 | 1.869 | 0.254 | 35 | 0.003 |
|  | Phgdh | 3.552 | 1 | 3.552 | 4.047 | 0.058 | 0.168 | 4.047 | 0.482 | 10 | 0.001 |
|  | Pla2g6 | 3.006 | 1 | 3.006 | 4.809 | 0.041 | 0.202 | 4.809 | 0.548 | 7 | 0.001 |
|  | Plcb1 | 2.968 | 1 | 2.968 | 3.333 | 0.083 | 0.143 | 3.333 | 0.412 | 14 | 0.001 |
|  | Prodh | 0.838 | 1 | 0.838 | 0.782 | 0.387 | 0.038 | 0.782 | 0.134 | 46 | 0.004 |
|  | Shank2 | 1.270 | 1 | 1.270 | 1.222 | 0.282 | 0.058 | 1.222 | 0.183 | 39 | 0.004 |
|  | Slc17a7 | 0.663 | 1 | 0.663 | 0.661 | 0.426 | 0.032 | 0.661 | 0.121 | 48 | 0.004 |
|  | Slc1a1 | 2.470 | 1 | 2.470 | 2.519 | 0.128 | 0.112 | 2.519 | 0.327 | 25 | 0.002 |
|  | Slc1a2 | 4.238 | 1 | 4.238 | 4.859 | 0.039 | 0.195 | 4.859 | 0.555 | 6 | 0.001 |
|  | Slc1a3 | 5.728 | 1 | 5.728 | 7.276 | 0.014 | 0.267 | 7.276 | 0.728 | 2 | 0.000 |
|  | Slc38a1 | 4.962 | 1 | 4.962 | 6.024 | 0.023 | 0.231 | 6.024 | 0.646 | 3 | 0.000 |
|  | Slc6a1 | 4.114 | 1 | 4.114 | 5.028 | 0.036 | 0.201 | 5.028 | 0.569 | 4 | 0.000 |
|  | Slc6a11 | 3.368 | 1 | 3.368 | 3.601 | 0.072 | 0.153 | 3.601 | 0.439 | 12 | 0.001 |
|  | Snca | 1.716 | 1 | 1.716 | 1.746 | 0.201 | 0.080 | 1.746 | 0.242 | 38 | 0.003 |
|  | Srr | 2.741 | 1 | 2.741 | 2.749 | 0.113 | 0.121 | 2.749 | 0.352 | 21 | 0.002 |
| Age * Diet | Abat | 0.144 | 1 | 0.144 | 0.154 | 0.699 | 0.008 | 0.154 | 0.066 | 26 | 0.002 |
|  | Adora1 | 0.557 | 1 | 0.557 | 0.544 | 0.469 | 0.026 | 0.544 | 0.108 | 9 | 0.001 |
|  | Aldh5a1 | 0.269 | 1 | 0.269 | 0.265 | 0.612 | 0.013 | 0.265 | 0.078 | 19 | 0.002 |
|  | App | 0.020 | 1 | 0.020 | 0.020 | 0.889 | 0.001 | 0.020 | 0.052 | 42 | 0.004 |
|  | Cdk5r1 | 0.003 | 1 | 0.003 | 0.002 | 0.962 | 0.000 | 0.002 | 0.050 | 11 | 0.001 |
|  | Dlg4 | 0.206 | 1 | 0.206 | 0.185 | 0.672 | 0.009 | 0.185 | 0.069 | 3 | 0.000 |
|  | Gabbr1 | 0.824 | 1 | 0.824 | 0.882 | 0.359 | 0.042 | 0.882 | 0.145 | 6 | 0.001 |
|  | Gabbr2 | 0.328 | 1 | 0.328 | 0.361 | 0.554 | 0.018 | 0.361 | 0.088 | 12 | 0.001 |
|  | Gabra1 | 0.059 | 1 | 0.059 | 0.062 | 0.806 | 0.003 | 0.062 | 0.056 | 36 | 0.003 |
|  | Gabra2 | 0.325 | 1 | 0.325 | 0.318 | 0.579 | 0.016 | 0.318 | 0.084 | 14 | 0.001 |
|  | Gabra4 | 0.005 | 1 | 0.005 | 0.006 | 0.940 | 0.000 | 0.006 | 0.051 | 52 | 0.005 |
|  | Gabra5 | 0.037 | 1 | 0.037 | 0.041 | 0.841 | 0.002 | 0.041 | 0.054 | 41 | 0.004 |
|  | Gabrb1 | 1.226 | 1 | 1.226 | 1.202 | 0.286 | 0.057 | 1.202 | 0.181 | 5 | 0.000 |
|  | Gabrb3 | 0.264 | 1 | 0.264 | 0.245 | 0.626 | 0.012 | 0.245 | 0.076 | 20 | 0.002 |
|  | Gabrg1 | 0.046 | 1 | 0.046 | 0.058 | 0.812 | 0.003 | 0.058 | 0.056 | 38 | 0.003 |
|  | Gabrg2 | 0.006 | 1 | 0.006 | 0.006 | 0.938 | 0.000 | 0.006 | 0.051 | 51 | 0.005 |
|  | Gabrg3 | 0.154 | 1 | 0.154 | 0.203 | 0.658 | 0.011 | 0.203 | 0.071 | 24 | 0.002 |
|  | Gad1 | 0.007 | 1 | 0.007 | 0.007 | 0.934 | 0.000 | 0.007 | 0.051 | 50 | 0.005 |
|  | Gls | 0.060 | 1 | 0.060 | 0.061 | 0.807 | 0.003 | 0.061 | 0.056 | 37 | 0.003 |
|  | Glul | 1.707 | 1 | 1.707 | 1.610 | 0.219 | 0.075 | 1.610 | 0.227 | 2 | 0.000 |
|  | Gnai1 | 0.096 | 1 | 0.096 | 0.095 | 0.761 | 0.005 | 0.095 | 0.060 | 32 | 0.003 |
|  | Gnaq | 0.005 | 1 | 0.005 | 0.005 | 0.943 | 0.000 | 0.005 | 0.051 | 53 | 0.005 |
|  | Gphn | 0.014 | 1 | 0.014 | 0.014 | 0.906 | 0.001 | 0.014 | 0.051 | 43 | 0.004 |
|  | Gria1 | 0.001 | 1 | 0.001 | 0.001 | 0.970 | 0.000 | 0.001 | 0.050 | 55 | 0.005 |
|  | Gria2 | 0.185 | 1 | 0.185 | 0.220 | 0.644 | 0.011 | 0.220 | 0.073 | 22 | 0.002 |
|  | Gria3 | 0.006 | 1 | 0.006 | 0.007 | 0.933 | 0.000 | 0.007 | 0.051 | 49 | 0.004 |
|  | Gria4 | 0.086 | 1 | 0.086 | 0.102 | 0.753 | 0.005 | 0.102 | 0.061 | 31 | 0.003 |
|  | Grik1 | 0.011 | 1 | 0.011 | 0.011 | 0.918 | 0.001 | 0.011 | 0.051 | 45 | 0.004 |
|  | Grik2 | 0.011 | 1 | 0.011 | 0.012 | 0.915 | 0.001 | 0.012 | 0.051 | 44 | 0.004 |
|  | Grik5 | 0.281 | 1 | 0.281 | 0.280 | 0.603 | 0.014 | 0.280 | 0.080 | 17 | 0.002 |
|  | Grin1 | 0.011 | 1 | 0.011 | 0.010 | 0.920 | 0.001 | 0.010 | 0.051 | 46 | 0.004 |
|  | Grin2a | 0.080 | 1 | 0.080 | 0.083 | 0.776 | 0.004 | 0.083 | 0.059 | 35 | 0.003 |
|  | Grin2b | 0.041 | 1 | 0.041 | 0.042 | 0.839 | 0.002 | 0.042 | 0.054 | 1 | 0.000 |
|  | Grm3 | 0.004 | 1 | 0.004 | 0.005 | 0.947 | 0.000 | 0.005 | 0.050 | 54 | 0.005 |
|  | Grm5 | 0.006 | 1 | 0.006 | 0.007 | 0.932 | 0.000 | 0.007 | 0.051 | 47 | 0.004 |
|  | Grm7 | 0.126 | 1 | 0.126 | 0.139 | 0.713 | 0.007 | 0.139 | 0.065 | 27 | 0.002 |
|  | Homer1 | 0.102 | 1 | 0.102 | 0.110 | 0.743 | 0.005 | 0.110 | 0.062 | 30 | 0.003 |
|  | Itpr1 | 0.007 | 1 | 0.007 | 0.007 | 0.933 | 0.000 | 0.007 | 0.051 | 48 | 0.004 |
|  | Mapk1 | 0.254 | 1 | 0.254 | 0.290 | 0.596 | 0.014 | 0.290 | 0.081 | 16 | 0.001 |
|  | Nsf | 0.044 | 1 | 0.044 | 0.056 | 0.816 | 0.003 | 0.056 | 0.056 | 39 | 0.004 |
|  | P2rx7 | 0.108 | 1 | 0.108 | 0.168 | 0.687 | 0.009 | 0.168 | 0.067 | 25 | 0.002 |
|  | Phgdh | 0.080 | 1 | 0.080 | 0.091 | 0.766 | 0.005 | 0.091 | 0.060 | 33 | 0.003 |
|  | Pla2g6 | 0.224 | 1 | 0.224 | 0.358 | 0.557 | 0.018 | 0.358 | 0.088 | 13 | 0.001 |
|  | Plcb1 | 0.108 | 1 | 0.108 | 0.121 | 0.731 | 0.006 | 0.121 | 0.063 | 29 | 0.003 |
|  | Prodh | 0.796 | 1 | 0.796 | 0.743 | 0.399 | 0.036 | 0.743 | 0.130 | 7 | 0.001 |
|  | Shank2 | 0.089 | 1 | 0.089 | 0.086 | 0.772 | 0.004 | 0.086 | 0.059 | 34 | 0.003 |
|  | Slc17a7 | 0.469 | 1 | 0.469 | 0.468 | 0.502 | 0.023 | 0.468 | 0.100 | 10 | 0.001 |
|  | Slc1a1 | 0.210 | 1 | 0.210 | 0.214 | 0.649 | 0.011 | 0.214 | 0.073 | 23 | 0.002 |
|  | Slc1a2 | 1.080 | 1 | 1.080 | 1.238 | 0.279 | 0.058 | 1.238 | 0.185 | 4 | 0.000 |
|  | Slc1a3 | 0.101 | 1 | 0.101 | 0.128 | 0.724 | 0.006 | 0.128 | 0.063 | 28 | 0.003 |
|  | Slc38a1 | 0.230 | 1 | 0.230 | 0.279 | 0.603 | 0.014 | 0.279 | 0.079 | 18 | 0.002 |
|  | Slc6a1 | 0.199 | 1 | 0.199 | 0.243 | 0.627 | 0.012 | 0.243 | 0.076 | 21 | 0.002 |
|  | Slc6a11 | 0.622 | 1 | 0.622 | 0.665 | 0.424 | 0.032 | 0.665 | 0.121 | 8 | 0.001 |
|  | Snca | 0.298 | 1 | 0.298 | 0.303 | 0.588 | 0.015 | 0.303 | 0.082 | 15 | 0.001 |
|  | Srr | 0.051 | 1 | 0.051 | 0.051 | 0.823 | 0.003 | 0.051 | 0.055 | 40 | 0.004 |

Table S8: Statisical summary for CA3 gene expression.
